# Regulation of the mechanoresponsive *Neat1* and PSPC1 by substrate stiffness in TGF-β1-induced renal progenitor cell fate

**DOI:** 10.1186/s12929-025-01196-w

**Published:** 2025-11-17

**Authors:** Hsiao-Ning Huang, Lun-Wei Lee, Cheng-Hsiang Kuo, Tzyy Yue Wong, Wen-Tai Chiu, Ming-Jer Tang

**Affiliations:** 1 Department of Physiology, College of Medicine, National Cheng Kung University, 1 University Rd., Tainan, 70101 Taiwan; 2 International Center for Wound Repair and Regeneration, National Cheng Kung University, 1 University Rd., Tainan, 70101 Taiwan; 3 Department of Biomedical Engineering, College of Engineering, National Cheng Kung University, 1 University Rd., Tainan, 70101 Taiwan; 4 Institute of Basic Medical Sciences, College of Medicine, National Cheng Kung University, 1 University Rd., Tainan, 70101 Taiwan; 5 Medical Device Innovation Center, National Cheng Kung University, Tainan, 70101 Taiwan

**Keywords:** PSPC1, *Neat1*, Matrix stiffness, Mouse kidney progenitor cell, TGF-β1, Renal fibrosis

## Abstract

**Background:**

Physical differences between acute kidney injury and chronic kidney disease, particularly in matrix stiffness, may influence mesenchymal stem cells to promote either regeneration or fibrosis; however, the underlying mechanisms remain unclear. Here, we investigate the role of paraspeckles and the long non-coding RNA *Neat1* in TGF-β1-induced stem cell fate determination.

**Methods:**

Mouse kidney progenitor cells (MKPCs) were cultured on stiff (collagen-coated dishes) and soft (type I collagen gel) matrices and treated with TGF-β1. RNA sequencing and subsequent bioinformatic analyses were performed to identify transcriptional differences between cells on stiff and soft matrices under TGF-β1 stimulation. Western-blotting and qPCR were used to quantify target proteins and RNA levels. Immunofluorescence staining and RNA fluorescence in situ hybridization were conducted to examine the subcellular localization of proteins and RNAs. Loss-of-function and gain-of-function experiments were performed using siRNA, shRNA, pharmacological inhibitors and expression vector.

**Results:**

We found that TGF-β1 induced MKPC differentiation into myofibroblasts on stiff matrices or endothelial-like cells on soft matrices. Matrix stiffness regulated PSPC1 and *Neat1* to trigger either TGF-β1-induced transdifferentiation into myofibroblasts or angiogenesis on soft collagen gels. Stiff matrices increased the expression levels of *Neat1* and PSPC1, whereas soft matrices reduced their expressions. Knockdown of PSPC1 impaired myofibroblast differentiation on stiff matrices and partially reduced angiogenesis on soft matrices. On stiff matrices, TGF-β1 markedly reduced *Neat1* levels, potentially releasing PSPC1 to interact with pSmad2/3 and activate EMT-related gene expression, thereby promoting myofibroblast activation. Furthermore, we identified two mechanosensory pathways that PSPC1 and *Neat1* responded to mechanical signals via β1-integrin-YAP and Piezo1 pathways.

**Conclusions:**

This study links mechano-regulation of paraspeckle complex to TGF-β1-induced renal mesenchymal stem cell fate, providing insights into mechanotransduction and nuclear signaling in kidney fibrosis and regeneration.

**Supplementary Information:**

The online version contains supplementary material available at 10.1186/s12929-025-01196-w.

## Background

The control of stem cell (SC) fate during tissue repair and regeneration has been a critical issue. In acute kidney injury (AKI), mesenchymal stem cells (MSCs), which are pluripotent SCs, have demonstrated benefits for kidney therapy and regeneration [1]. These cells have the ability to differentiate into renal tubules or neovasculature to facilitate the repair of renal ischemia–reperfusion injury [2]. However, in chronic kidney disease (CKD), where myofibroblasts play an essential role in promoting tubulointerstitial fibrosis, it has been reported that resident MSCs serve as one of the origins of myofibroblasts during kidney fibrosis [3]. Since MSCs may facilitate kidney regeneration during AKI, but contribute to tubulointerstitial fibrosis during CKD, what are the causes of the determination of MSC fate is a critical issue. Matrix stiffness is known to play a crucial role in regulating several cellular behaviors, such as cell survival, proliferation, differentiation, and migration [4–6]. Under physiological conditions, tissue stiffness is relatively low, with a soft matrix potentially acting as a cell cycle inhibitor. Conversely, increased tissue stiffness has been shown to promote cell proliferation both *in vivo* and *in vitro* and is highly relative to poor pathological process [7]. More importantly, our previous research has shown that high matrix stiffness facilitates TGF-β1-induced epithelial mesenchymal transdifferentiation (EMT) which may contribute to accelerating the progression of organ fibrosis, while low matrix stiffness inhibits cell dedifferentiation and prevents TGF-β1-induced EMT [6, 8]. These findings suggest that the differentiation direction of MSCs is potentially regulated by matrix stiffness, leading to different pathological outcomes.

Paraspeckle complex is an RNA–protein nuclear bodies specific in mammals, formed by paraspeckle component 1 (PSPC1), long non-coding RNA (lncRNA) *Neat1* (nuclear paraspeckle assembly transcript 1) and other over 40 associated proteins [9–11]. PSPC1 has been reported to play a crucial role in determination of TGF-β1-induced cell fate by acting as a cofactor that promotes EMT and stemness or cell growth inhibition and differentiation through TGF-β1-Smad2/3 signaling [12]. In contrast, PSPC1-deficient cells treated with TGF-β1 exhibit cell apoptosis or growth inhibition [12]. On the other hand, there are two *Neat1* isoforms: the short *Neat1* (3700 nt) and the long *Neat1_2* (23,000 nt). Both isoforms participate in maintaining paraspeckles formation, though the longer *Neat1_2* plays a more critical role in paraspeckle assembly [13]. Previous studies show that *Neat1* is sensitive to mechanical stimuli (e.g. matrix stiffness) and involved in regulating osteoblast function through paraspeckle-dependent mechanism [14, 15]. These findings suggest that *Neat1*, associated with PSPC1, may mediate the differentiation capacity of MSCs in response to different matrix stiffness upon TGF-β1 stimulation. Therefore, we hypothesized that paraspeckle components, particularly *Neat1* and PSPC1, were involved in modulating the matrix stiffness-induced switch in MSC specification through mechanotransduction pathway.

To examine our hypothesis, we utilized mouse kidney progenitor cells (MKPCs) isolated from mouse kidney interstitial tissue, which exhibit multilineage differentiation potential, including differentiation into osteoblasts and endothelial cells [16]. Furthermore, we demonstrated that MKPCs contribute to accelerate renal regeneration during renal ischemic injury, supporting their multipotent SC capacity [16, 17]. In this study, we found that MKPCs differentiate into angiogenetic or myofibrogenic lineages in response to different matrix stiffnesses when exposed to TGF-β1. A stiff matrix promoted the differentiation of MKPCs into myofibroblasts in response to TGF-β1, while a soft collagen gel facilitated TGF-β1-induced angiogenesis. These two distinct responses were mediated by the unique nuclear mechanics of the mechanosensitive components *Neat1* and PSPC1. We explore the mechanotransduction mechanisms linked with the changes of paraspeckles whereby matrix stiffness triggers differentiation of MKPCs into either myofibroblast activation or angiogenesis under TGF-β1 stimulation.

## Methods

### Cell isolation and treatment

Mouse kidney progenitor cells (MKPCs) were isolated from *Myh9*-targeted mutant mice carrying an emGFP transgene tagged with the *Myh9* sequence [16]. Briefly, kidneys were dissected and enzymatically digested with 0.3% collagenase and 0.3% trypsin at 37 °C for 30 min. The digested suspension was filtered through a 100 µm mesh. The filtrate was further homogenized using a Dounce Tissue Homogenizer and filtered through a 40 µm mesh to remove cell aggregates, resulting in a single-cell suspension. Green fluorescent protein (GFP)-positive cells were isolated using fluorescence-activated cell sorting (FACS) on a FACSAria Cell Sorter (BD Biosciences, San Diego, USA). MKPCs were maintained as a cell line in Dulbecco’s modified Eagle’s medium, low-glucose (DMEM-LG, Thermo Scientific, Cat. No. 31600034) supplemented with 100 IU/ml penicillin (Sigma, Cat. No. P3032), 100 μg/ml streptomycin (Sigma, Cat. No. S6501), and 10% cosmic calf serum (CCS, Hyclone, Cat. No.SH30087.03) at 37 °C in the presence of 5% CO_2_. Cells in early passages (fewer than 10 after thawing) were used in this study. MKPCs were treated with 10 ng/ml human TGF-β1 recombinant protein (PreproTech, Cat. No. 100-21C) for 16 h for all experiments.

### Collagen gel

The concentration of collagen gel is 0.9 mg/ml, containing 5.7X concentrated DMEM-LG, 2.5% NaHCO_3_ (Sigma, Cat. No. S5761), 0.1 M HEPES, pH8.5 (Sigma, Cat. No. H3375), 0.17 M CaCl_2_ (Sigma, Cat. No. C4901), 1 N NaOH (Sigma, Cat. No. S8045), and 1% Collagen type I from the rat tail (Corning Life Science, Cat. No. 354236).

### Polyacrylamide (PA) gel with different stiffness

PA gels with varying stiffness were first cast between a SIGMACOTE^®^ (Sigma, Cat. No. SL2)-activated glass coverslip and a 3-amino-propyl-trimethoxysilane (Sigma, Cat. No. 281778)-activated glass coverslip. Different gel stiffnesses were achieved by mixing varying concentrations of acrylamide (Sigma, Cat. No. A8887) and bis-acrylamide (Sigma, Cat. No. 146072) with 30% acrylic acid, 10% ammonium persulfate (Sigma, Cat. No. A3678), and TEMED (Sigma, Cat. No. T9281). After polymerization, the PA gel surface was activated using EDC [1-ethyl-3-[3-dimethylaminopropyl] carbodiimide hydrochloride] (Sigma, Cat. No. 341006). Following extensive washing with 0.1 M MES [2-(N-morpholino) ethanesulfonic acid] (Sigma, Cat. No. M3671), 100 μg/ml type I collagen (Corning Life Science, Cat. No. 354236) in 0.1 M MES was applied to the PA gel and incubated overnight at 4 °C. Finally, PA gels were thoroughly rinsed with PBS and soaked in culture medium before use. The mechanical properties of the PA gels from each polymerization batch were verified using atomic force microscopy (AFM).

### RNA extraction and quantitative real-time polymerase chain reaction (qRT–PCR)

Total RNA was extracted using the RNeasy^®^ Mini kit (Qiagen, Cat. No. 74106). cDNA synthesis was performed with 1 µg of total RNA using SuperScript IV reverse transcriptase (Life Technologies, Cat. No.18090050) and random hexamer primers (Promega, Cat. No. C1181). qRT-PCR was conducted in triplicate on a StepOne^™^ real-time PCR instrument, and the data were analyzed using StepOne™ Software v2.0 (Applied Biosystems, Life Technologies).

### RNA sequencing and data analysis

Total RNA was extracted using the RNeasy^®^ Mini kit (Qiagen, Cat. No. 74106), and RNA quality was assessed using the SimpliNanoTM—Biochrom Spectrophotometers (Biochrom, MA, USA). RNA-seq libraries were prepared manually from the isolated total RNA using the KAPA mRNA HyperPrep Kit (KAPA Biosystems, Roche, Basel, Switzerland) according to the manufacturer’s protocol. The preparation involved isolating mRNA from total RNA using magnetic oligo-dT beads, followed by steps including fragmentation, first-strand cDNA synthesis, second-strand synthesis with A-tailing, dUTP incorporation, dsDNA adapter ligation, and the size selection of cDNA fragments. Library amplification was performed using KAPA HiFi HotStart ReadyMix and library amplification primers, ensuring strand-specific sequencing with dUTP-marked non-amplified strands. PCR products were subsequently purified, and library quality was evaluated using the Qubit@ 2.0 Fluorometer and Agilent Bioanalyzer 2100 system. Sequencing was performed on the Illumina NovaSeq6000 platform, generating 150 bp paired-end reads.

Raw sequenced reads were produced via CASAVA base calling and stored in FASTQ format. Quality assessment was conducted using FastQC and MultiQC. The raw paired-end reads were processed with Trimmomatic to remove low-quality reads, trim adaptor sequences, and eliminate poor-quality bases, resulting in high-quality data for downstream analysis. Read pairs from each sample were aligned to the reference genome (mm9/NCBIM37.67) using HISAT2, followed by read counting with FeatureCounts.

Gene expression analysis included normalization using the “Trimmed Mean of M-values” (TMM) without a biological duplicate and “Relative Log Expression” (RLE) with a biological duplicate. Differentially expressed genes (DEGs) analysis was carried out using DEGseq (without replicates) and DESeq2 (with replicates). *P*-values were adjusted using the Benjamini–Hochberg method for false discovery rate (FDR) control. Genes were considered differentially expressed if they had an FDR value of < 0.05 and a log2 fold-change >  ± 0.6 unless otherwise noted. GO and KEGG pathway enrichment analyses were performed using clusterProfiler, and the DOSE package was utilized for Disease Ontology (DO) mapping. Gene set enrichment analysis (GSEA) was conducted with the Molecular Signatures Database (MSigDB) with 1,000 permutations. A protein–protein interaction (PPI) network was constructed based on DEGs using STRINGdb. Finally, Weighted Gene Co-expression Network Analysis (WGCNA) was performed to generate a co-expression network based on the correlation coefficient of the expression pattern using the WGCNA package in R.

### Cell lysates collection and Western blots analysis

Cell lysates were harvested using RIPA buffer: including 20 mM Tris (pH 7.4), 300 nM NaCl, 10 mM EDTA, 2 mM EGTA, 2% Triton X-100, 2% sodium deoxycholate, 1% SDS, 1 mM sodium orthovanadate (Na_3_VO_4_), 1 mM phenylmethanesulfomyl fluoride (PMSF), and protease inhibitor cocktail (Sigma, Cat. No. P8340). Protein concentrations in the samples were quantified using the Lowry assay and bovine serum albumin (BSA) as the standard. Protein lysates (30 μg) were seperated by SDS-PAGE and electroblotted onto a polyvinylidene difluoride (PVDF) membrane (Merck Millipore, Cat. No. IPVH00010). The PVDF membranes were blocked with 4% fat-free dry milk in TBST for 1 h at room temperature, followed by incubation with specific primary antibodies. Primary antibodies used for western blot including PSPC1 (Sigma, HPA038904); SFPQ (Sigma, HPA054094); NONO (Sigma, HPA054689); β1-integrin (BD Biosciences, 559883), α-SMA (Sigma, 5228), FN1, YAP (Cell Signaling, 14074), Piezo1 (Sigma, NBP1-78537) and GAPDH (Glyceraldehyde 3-phosphate dehydrogenase) (Proteintech, 60004–1-Ig). Immunocomplexes were detected using horseradish peroxidase-conjugated secondary antibodies and visualized via fluorography using an enhanced chemiluminescence (ECL) detection kit (Thermo Scientific, Cat. No. 34580).

### Plasmid constructs, short hairpin RNA, and lentiviral infections

The full-length PSPC1 cDNA was cloned into pAS4.1w.Pbsd-aOn plasmids. The short hairpin RNAs (shRNAs) targeting mouse PSPC1 and β1 integrin were obtained from the National RNAi Core Facility Platform of Academia Sinica. Lentiviral preparation and virus infection were performed according to the protocol from the National RNAi Core Facility Platform. Briefly, pLKO.1 with shRNA, pMD.G, and pCMV-ΔR8.91 were transfected into 293 T cells for lentiviral packaging. The viral supernatants were collected and used to infect MKPCs. Infected cells were selected with 2 μg/ml puromycin (Sigma, Cat. No. P8833) or blasticidine (Sigma, Cat. No. 15205) for 1 week. Single colonies were isolated and analyzed for target protein levels via western blotting. The shRNA sequences are provided in Table S1.

### Immunofluorescence staining

Cells were fixed with 4% paraformaldehyde for immunocytochemistry and permeabilized with 0.5% Triton X-100. Following fixation, cells were incubated with SuperBlock® blocking buffer (Thermo Scientific,  Cat. No. 37515) for 1 h and followed by incubation with specific primary antibodies overnight. Primary antibodies used for immunofluorescence staining including PSPC1 (Sigma, HPA038904) and Smad2 (Cell Signaling, #5339). After washing with PBS, cells were incubated with secondary antibodies conjugated with fluorescent markers (Molecular Probes) for anti-mouse or rabbit IgG, along with 10 μg/ml Hoechst 33258 (Sigma-Aldrich) for 1 h.

### Microscopy and image analysis

All fluorescence images were captured using an FV3000 confocal microscope (Olympus) equipped with a 40X oil-immersion objective lens (numerical aperture: 1.30). Imaging was performed at room temperature, with oil as the immersion medium. Fluorescence signals were detected using Alexa-conjugated secondary antibodies. Images were captured and recorded using FV31S-SW acquisition software. Subsequent image processing and analysis were performed using Image Pro Plus software.

### In situ hybridization

Stellaris^®^ *NEAT1* RNA FISH probes targeting the *Neat1*_1 isoform (VSMF-3028–5, Quasar® 590-conjugated) and *Neat1*_2 isoform (VSMF-3032–5, Quasar® 590-conjugated) were purchased from LGC Biosearch Technologies. Cell preparation, hybridization, and mounting were carried out following the Stellaris^®^ RNA-FISH Probes manual. In brief, cells were seeded onto circular coverslips in a 6-well plate and allowed to adhere for 1 day. They were then fixed with 4% paraformaldehyde and permeabilized with 0.5% Triton X-100. Hybridization was conducted overnight at 37 °C in a humidifying chamber. For co-immuno-FISH experiments, hybridization was performed as described above, followed by incubation in SuperBlock^®^ blocking buffer (Thermo Scientific, Cat. No. 37515) for 1 h at room temperature. Cells were then incubated with primary antibodies overnight at 4 °C. After rinsing with PBS, cells were incubated with Alexa 594 or Alexa 647-conjugated antibodies (Thermo Fisher Scientific-Invitrogen) for 1 h at room temperature. Samples were mounted using Fluoroshield Mounting Medium with DAPI (Abcam, Cambridge, UK). *Neat1* signal counts and areas were quantified using Image Pro Plus software.

### Statistical analysis

All results are presented as the mean ± SEM. A two-tailed Student’s *t*-test was used to compare differences between two groups, and the one-way analysis of variance (ANOVA) was applied for comparisons involving more than two independent groups. GraphPad Prism was used for the statistical analyses, with significance set at *p < 0.05; **p < 0.01; ***p < 0.001.

## Results

### TGF-β1-induced MKPC cell transdifferentiation is determined by matrix rigidity

**Fig. 1:**
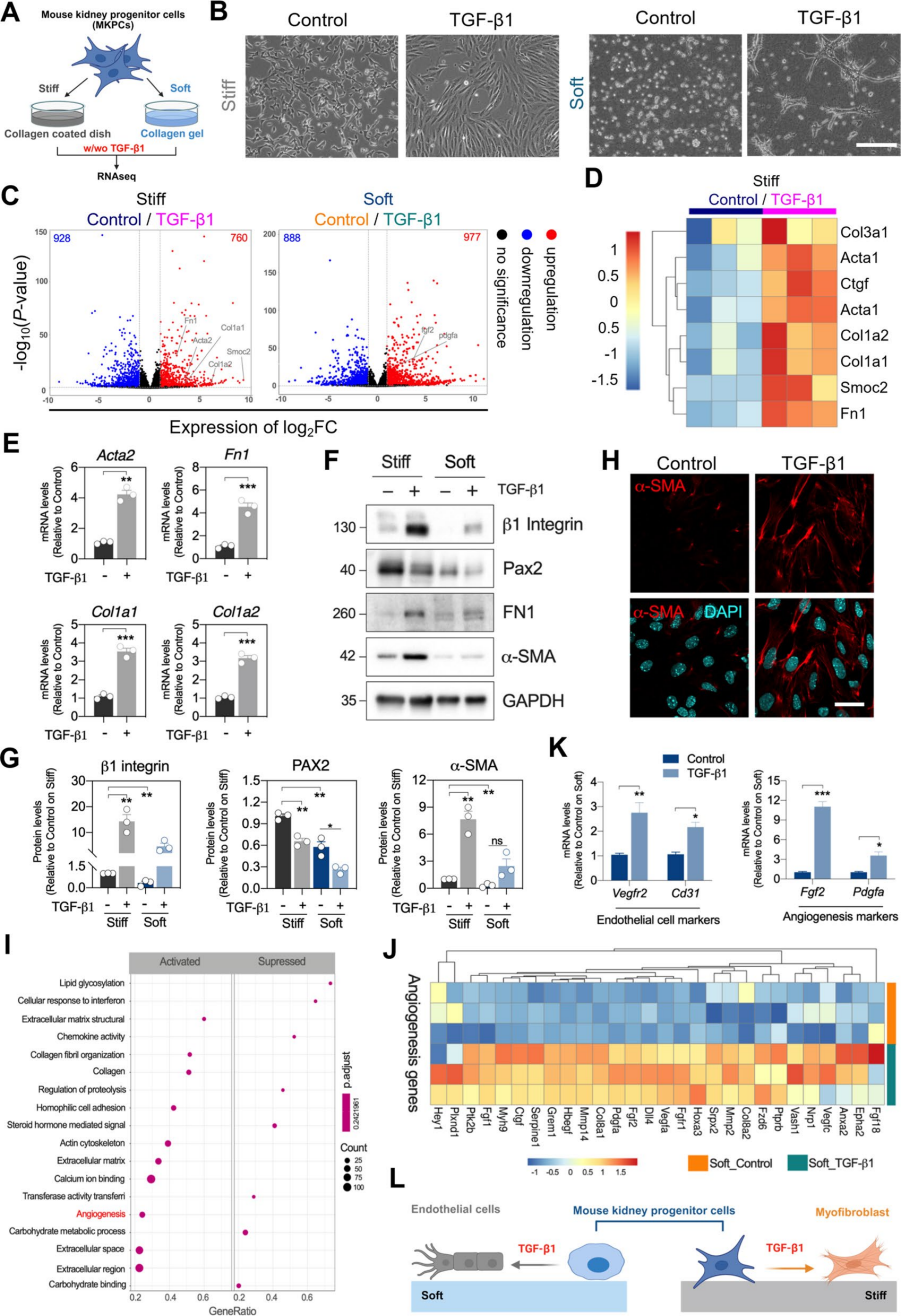
TGF-β1-treated mouse kidney progenitor cells (MKPCs) on stiff or soft matrices developed into different cell fates. **A** Diagram of the experimental design where MKPCs cultured on type I collagen (Col)-coated dishes (stiff matrix) or Col gel (soft matrix) and treated with or without TGF-β1. **B** Representative phase-contrast images of cell morphology of MKPCs after TGF-β1 treatment on stiff (left) and soft matrices (right). Scale bar: 200 μm. **C** Volcano plots showing differential gene expression in control versus TGF-β1-treated MKPCs on stiff (left) or soft matrices (right). Selected candidate genes are highlighted. Horizontal and vertical dashed lines represent the false discovery rate (FDR, 0.05) and log_2_ fold change (± 1.5) cut-offs, respectively. Number of Genes that are significantly up- or down-regulated are shown in the right or left top corners, respectively. **D** Heatmap displaying the expression of selected myofibroblast activation marker genes in control or TGF-β1-treated MKPCs on stiff matrix. Data are presented as log_2_ normalized counts. **E** Gene expression analysis of myofibroblast marker genes (*Acta2, Fn1, Col1a1 and Col1a2*) in TGF-β1-treated MKPCs on stiff matrix, analyzed by qRT-PCR and normalized to *Gapdh*. **F** Representative Western blot images showing protein levels of β1-integrin, Pax2, fibronectin 1 (FN1), and α-SMA, with GAPDH used as the internal control. **G** Quantification of protein levels of β1-integrin, Pax2 and α-SMA on stiff and soft matrices with TGF-β1 treatment. **H** Representative immunofluorescence images displaying the distribution of α-SMA (red) and nuclei (cyan) in TGF-β1-treated MKPCs on stiff matrix. Scale bar: 30 μm. **(I)** Gene Ontology (GO) term enrichment analysis of the top 10 enriched pathways in TGF-β1-treated MKPCs on soft matrix. The adjusted p value (P.adjust) represents pathway enrichment, while the GeneRatio denotes the percentage of target genes in the enriched pathway. Dots size is proportional to the number of differentially expressed genes (DEG) in the pathway. **J** Heatmap showing gene expression related to angiogenesis in TGF-β1-treated MKPCs on soft matrix. **K** Gene expression levels of endothelial marker genes (*Vegfr2* and *Cd31*) and angiogenesis marker genes (*Fgf2 and Pdgfa*) in TGF-β1-treated MKPCs on soft matrix, analyzed by qRT-PCR. **L** Schematic diagram illustrating the differentiation of TGF-β1-treated MKPCs into myofibroblasts on stiff matrix and into endothelial cells on soft matrix. Data are presented as individual points with mean ± S.E.M. from at least three independent experiments. Statistical analysis was performed using a two-tailed unpaired *t*-test. *P < 0.05; **P < 0.01; ***P < 0.001

MKPCs derived from *Myh9*-targeted mice demonstrate multipotent abilities both *in vitro* and *in vivo* [16, 17]. To investigate whether MKPCs alter their behaviors in response to different matrix stiffnesses, we seeded MKPCs on the top of both stiff type I-collagen (Col) coated dishes (rigidity over gigapascal) (GPa) and soft Col gel matrices (rigidity approximately 40 Pa) with TGF-β1 treatments, ensuring mechanical stimulation was applied from the basal region. We then employed RNA-seq to profile the transcriptome of MKPCs (Fig. 1A). Morphologically, MKPCs cultured on a stiff matrix displayed a long spindle-shaped phenotype (Fig. 1B, left), whereas those on the soft matrix exhibited a tube-like morphology (Fig. 1B, right). Transcriptomic profiling using principal component analysis (PCA) to reveal distinct gene expression clusters across the groups demonstrates that both matrix stiffness and TGF-β1 induced global transcriptomic changes in MKPCs (Fig S1A). We further compared gene expression changes in the presence/absence of TGF-β1 on stiff and soft matrices showing with volcano plot (Fig. 1C). Notably, we found that the expressions of fibrosis-related genes, including *Acta2*, *Fn1* and *Col1a1*, were upregulated by TGF-β1 on a stiff matrix, whereas angiogenesis-related genes, such as *Fgf2* and *Pdgfa*, were upregulated by TGF-β1 on a soft matrix (Fig. 1C). These results revealed that TGF-β1-induced MKPC transdifferentiation toward fibrogenesis or angiogenesis depends on matrix stiffness.

The top 8 genes which were significantly upregulated by TGF-β1 on stiff matrices were highlighted in the heatmap (Fig. 1D). These upregulated genes are involved in extracellular matrix (ECM) remodeling and assembly that are critical for myofibroblast activation. To confirm the expressions of these fibrogenic genes, we verified that the mRNA levels of *Acta2*, *Fn1*, *Col1a1* and *Col1a2* were upregulated under the stiff conditions with TGF-β1 treatment (Fig. 1E). The protein expression and distribution of TGF-β1-mediated upregulated *Acta2,* a key gene for myofibroblast differentiation encoding α-SMA, were also validated in MKPCs (Fig. 1F‐1H). Interestingly, Pax2, a marker for multipotent MKPCs, was highly expressed in untreated cells but reduced by TGF-β1 treatment, indicating a transdifferentiation in MSCs (Fig. 1F and G). These findings suggested that TGF-β1 treatment under stiff conditions stimulates a fibrogenic program in MKPCs, promoting MKPCs transdifferentiation into myofibroblasts.

In contrast to the stiff matrix, TGF-β1-treated MKPCs seeded on a soft matrix exhibited distinct tubule morphology, showing a different cell fate (Fig. 1B, right). We performed gene ontology (GO) analysis according to the RNA-seq data to identify the potential pathways involved in the morphological change upon TGF-β1 treatment on soft matrices. We found that TGF-β1-treated MKPCs on the soft matrix showed a higher prominence in pathways related to angiogenesis, calcium ion binding, collagen fibril organization and actin cytoskeleton pathways (Fig. 1I). Notably, we observed robust increases in endothelial-related and angiogenesis-related genes in MKPCs with TGF-β1 on soft matrices (Fig. 1J). The upregulations of endothelial cell markers (*Vegfr2* and *Cd31*) and angiogenesis-related genes (*Fgf2* and *Pdgfa*) were further validated (Fig. 1K). Taken together, our data demonstrate that matrix stiffness critically regulates MSC fate determination in response to TGF-β1 treatment. Particularly, TGF-β1 drives MKPC transdifferentiation toward myofibroblast on stiff matrices, while it promotes differentiation into endothelial cells forming tubule-like structures on soft matrices, implying a mechanosensitive mechanism involved in controlling MSC fate determination (Fig. 1L).

### Paraspeckles and *Neat1* in MKPCs are mechanosensitive to matrix stiffness

**Fig. 2:**
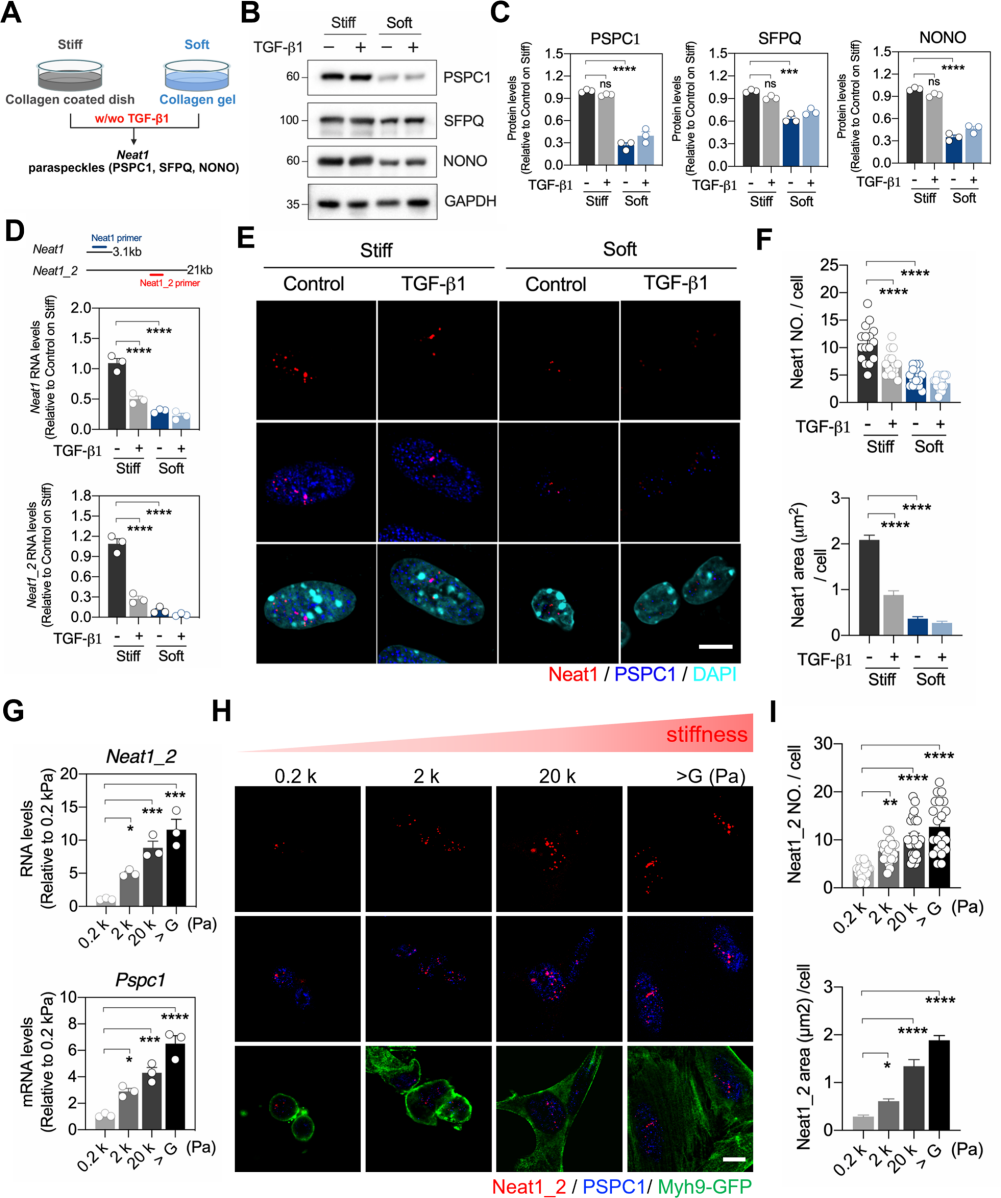
The expression of paraspeckles and *Neat1* were regulated by matrix stiffness and TGF-β1 in MKPCs.** A** Diagram of the experimental design where MKPCs were cultured on either Col-coated dishes or Col gel, treated with or without TGF-β1, and analyzed for the expression of paraspeckle components. **B** Representative Western blot images showing the protein levels of paraspeckles components (PSPC1, SFPQ, and NONO) with GAPDH used as the internal control. **C** Quantification of protein levels of SFPQ, PSPC1 and NONO on stiff and soft matrices with TGF-β1 treatment. **D** Gene expression of *Neat1* (shorter isoform) and *Neat1_2* (longer isoform) in TGF-β1-treated MKPCs on stiff or soft matrices, as evaluated by qRT-PCR. Expression levels were normalized to *Gapdh*. **E** Representative images from *in-situ* hybridization of *Neat1* (red) and immunofluorescence staining of PSPC1 (blue) in TGF-β1-treated MKPCs on stiff or soft matrices. Scale bar: 5 μm. **F** Quantification of the number and area of *Neat1* RNA in TGF-β1-treated MKPCs grown on stiff or soft matrices. **G** Gene expression analysis of *Neat1_2* and *Pspc1* RNA in MKPCs grown on substrates with various stiffness levels, assessed by qRT-PCR and normalized to *Gapdh*. **H** Representative images of *in-situ* hybridization of *Neat1_2* (red) and immunofluorescence staining of PSPC1 (blue) and Myh9-GFP (green) in TGF-β1-treated MKPCs on matrices of varying stiffness. Scale bar: 10 μm. **I** Quantification of the number and area of *Neat1_2* per cell in MKPCs cultured on matrices of varying stiffness. All data are presented as individual points with mean ± S.E.M. from at least three independent experiments. Two-way ANOVA was used for statistical analysis in (C), (D) and (F), and one-way ANOVA in (G) and (I), with Bonferroni multiple comparison tests. *P < 0.05; **P < 0.01; ***P < 0.001; ****P < 0.0001

Both PSPC1 and lncRNA *Neat1* are critical nuclear paraspeckle components involved in regulating various cellular behaviors, including TGF-β1-induced EMT, stemness or growth inhibition, and are considered mechanosensitive [14]. Our RNA-seq data also revealed that genes encoding paraspeckle components, including *Neat1*, *Pspc1*, *Sfpq*, and *Nono* were downregulated in MKPCs cultured on a soft matrix compared to those on a stiff matrix (Fig. S1B). To verify the expression of paraspeckle components, we examined the levels of *Neat1* and paraspeckle proteins in TGF-β1-treated MKPCs on stiff or soft matrix (Fig. 2A). As expected, the expression of paraspeckle complex proteins, including PSPC1, SFPQ, and NONO, were downregulated in the MKPCs on the soft matrix compared to the stiff matrix (Fig. 2B and C). Both *Neat1* and *Neat1_2* levels were significantly lower in MKPCs on the soft matrix compared to the stiff matrix (Fig. 2D). Using an *in-situ* hybridization approach with RNA probes recognizing regions common to both *Neat1* isoforms, or specific to *Neat1_2*, we observed that *Neat1* was predominantly expressed in the nucleus (Fig. 2E). Additionally, the number and area of *Neat1* and *Neat1_2* within the nucleus were also reduced on the soft matrix (Fig. 2E and F; Fig. S1C and S1D). Interestingly, TGF-β1 treatment in MKPCs cultured on stiff matrices markedly reduced *Neat1* expression, as well as the number and area of *Neat1* and *Neat1_2* (Fig. 2D and E), indicating that both matrix rigidity and TGF-β1 regulate *Neat1* expression. In addition, PSPC1 colocalized with TGF-β1-induced nuclear Smad2 on stiff matrices (Fig. S2), suggesting a potential role for PSPC1 in promoting fibrogenic differentiation. These findings provide evidence that *Neat1* and paraspeckle complex proteins in MKPCs are mechanosensitive in response to different matrix stiffness.

*Neat1_2*, the longer *Neat1* isoform, is crucial for paraspeckle assembly [13], we further examine the *Neat1_2* expression on various matrix stiffness using polyacrylamide (PA) gels, which have been widely used to study the mechanical interactions between cells and the ECM [18]. We cultured MKPCs on PA gels with rigidities ranging from 0.2 kPa to over GPa. The expression levels *Neat1_2* and *Pspc1* were increased by substrate stiffness in a dose-dependent manner, accompanied by enhanced aggregation of *Neat1_2* (Fig. 2G‐I), reinforcing that the expression of *Neat1* and PSPC1 in MSCs were modulated by matrix stiffness.

### *Neat1* and PSPC1 are required for TGF-β1-induced MKPC fate determination in response to matrix rigidity

**Fig. 3:**
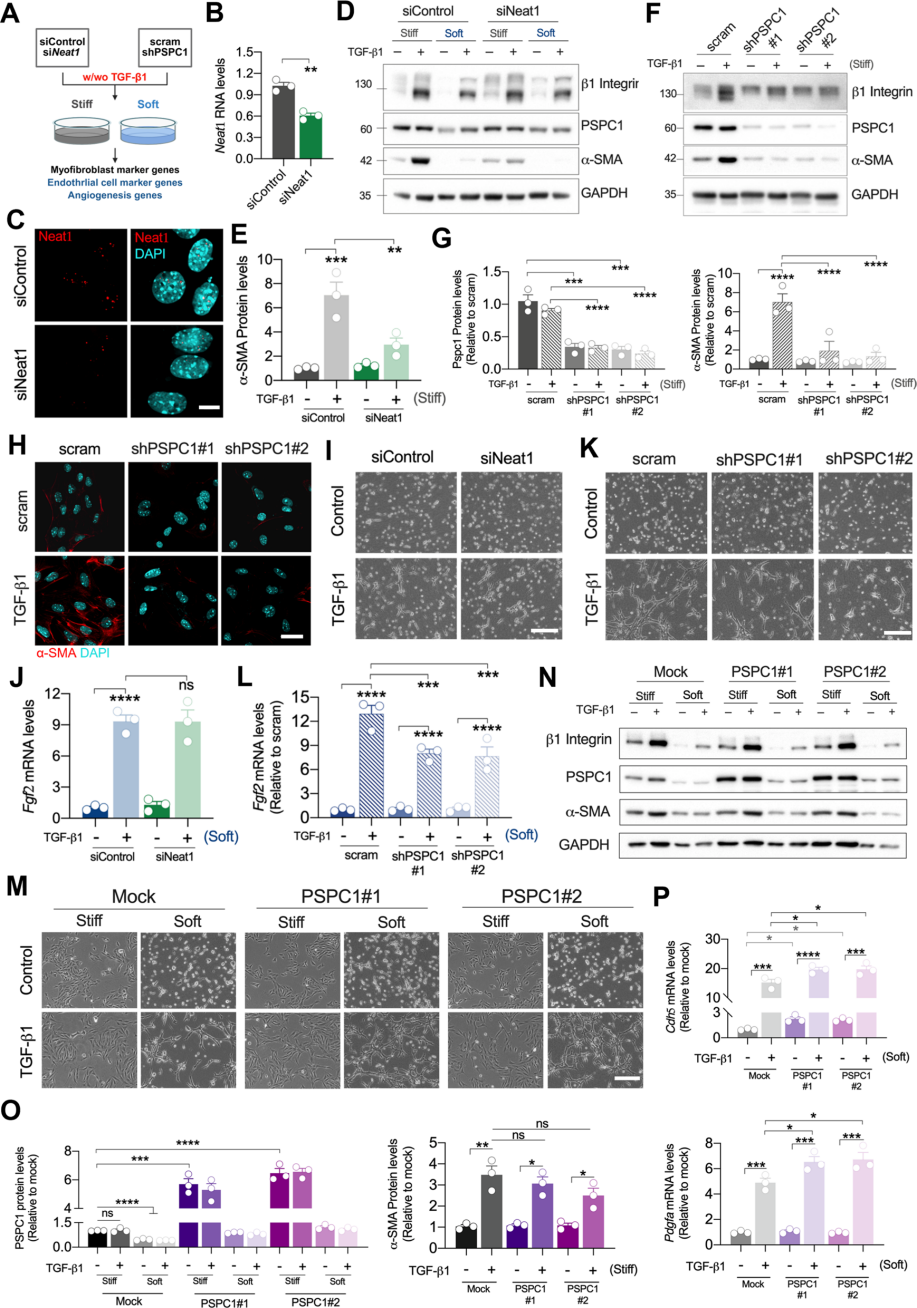
PSPC1 and *Neat1* were crucial for TGF-β1-induced cell fate determination in response to matrix stiffness. **A** Diagram of the experimental design of MKPCs transfected with siNeat1 or shPSPC1, treated with TGF-β1 treatment and cultured on stiff or soft matrices. **B** Gene expression analysis of *Neat1* in MKPCs on stiff matrix transfected with *siNeat1*, evaluated by qRT-PCR. **C** Representative images from *in-situ* hybridization of *Neat1* (red) and nuclei (cyan) in siControl- and si*Neat1*-treated MKPCs on stiff matrix. Scale bar: 20 μm. **D** Representative Western blot images of the protein levels of β1-integrin, PSPC1, and α-SMA in siControl- and si*Neat1*-treated MKPCs on stiff or soft matrix with TGF-β1 treatment using GAPDH as the internal control. **E** Quantification of protein levels of α-SMA in siControl- and si*Neat1*-treated MKPCs on stiff matrix with TGF-β1 treatment. **F** Representative Western blot images showing PSPC1, β1-intergrin, and α-SMA expressions in TGF-β1-treated MKPCs on stiff matrix infected with shPSPC1, using GAPDH as the internal control. **G** Quantification of PSPC1 and α-SMA protein levels in MKPCs treated with shPSPC1 and TGF-β1 on stiff matrix. **H** Representative immunofluorescence images showing α-SMA (red) and nuclei (cyan) in PSPC1-depleted MKPCs on stiff matrix. Scale bar: 30 μm. **I** Representative phase-contrast images of siControl- and si*Neat1*-treated MKPCs on soft matrix with TGF-β1 treatment. Scale bar: 200 μm. **J** Gene expression analysis of the angiogenesis marker *Fgf2* in siControl- and si*Neat1*-treated MKPCs on soft matrix with TGF-β1 treatment. **K** Representative phase-contrast images of scramble and shPSPC1-infected MKPCs treated with TGF-β1 on soft matrix. Scale bar: 200 μm. **L** Gene expression of the angiogenesis marker *Fgf2* in MKPCs infected with shPSPC1 on soft matrix as analyzed by qRT-PCR. **M** Representative phase-contrast images of mock and PSPC1-overexpressing MKPCs (PSPC1#1 and #2) treated with TGF-β1 on stiff or soft matrix. Scale bar: 200 μm. **N** Representative Western blot images of the protein levels of β1-integrin, PSPC1, and α-SMA in mock and PSPC1-overexpressing MKPCs on stiff or soft matrix with TGF-β1 treatment using GAPDH as the internal control. **O** Quantification of protein levels of PSPC1 and α-SMA in mock and PSPC1-overexpressing MKPCs. **P** Gene expression analysis of the angiogenesis markers *Cdh5* and *Pdgfα* in mock and PSPC1-overexpressing MKPCs on soft matrix with TGF-β1 treatment. Data are presented as individual points with mean ± S.E.M. from at least three independent experiments. Statistical analysis was performed using a two-tailed unpaired *t*-test (B) and two-way ANOVA (E, G, J, L, O, P) with Bonferroni multiple comparison tests. **P < 0.01; ***P < 0.001; ****P < 0.0001

To evaluate whether *Neat1* and PSPC1 regulate the determination of MKPC transdifferentiation on different matrix stiffness upon TGF-β1, we knockdowned *Neat1* and PSPC1 and examined the expressions of fibrogenic and angiogenic markers on stiff and soft matrices (Fig. 3A). The knockdown efficacy of si*Neat1* in MKPCs was confirmed at the RNA levels and immunofluorescence (Fig. 3B and C). While reduction of *Neat1* expression did not significantly alter α-SMA expression in MKPCs on a stiff matrix, it markedly downregulated TGF-β1-induced α-SMA upregulation (Fig. 3D and E). Besides, knockdown of PSPC1 significantly reduced α-SMA expression in TGF-β1-treated MKPCs on stiff matrices (Fig. 3F‐H). These results showed that depletion of *Neat1* or PSPC1 prohibited TGF-β1-induced upregulation of α-SMA on stiff substrates, implying that both *Neat1* and PSPC1 participate in promoting TGF-β1-induced MKPC transdifferentiation into myofibroblast on stiff matrices.

We previously demonstrated that both *Neat1* and PSPC1 in MKPCs were downregulated by culturing on soft matrices (Fig. 2B‐D). Further, we examined whether knockdown of *Neat1* or PSPC1 affects MKPC transdifferentiation into endothelial-like cells upon TGF-β1 treatment on soft matrices. However, knockdown of *Neat1* in MKPCs did not alter TGF-β1-induced tubule-like morphology or the expressions of endothelial markers and angiogenesis-related proteins (Fig. 3I and J; Fig. S3A and S3B). On the contrary, knockdown of PSPC1 suppressed the formation of tube-like structures, as well as decreased the expressions of endothelial cell markers (*Cdh5* and *Vegfr2*) and angiogenesis-related genes (*Fgf2* and *Pdgfa*) (Fig. 3K and L; Fig. S3C and S3D).

To validate the impact of PSPC1 on MKPC differentiation under stiff and soft matrix conditions, we overexpressed PSPC1 (PSPC1#1 and #2) in MKPCs and confirmed the overexpression efficiency (Fig. 3N and O). Although the PSPC1 level remained relatively low on soft matrices even after overexpression, it was still higher than that observed in the mock group under the same conditions (Fig. 3N and O). Upon TGF-β1 treatment, PSPC1 overexpression enhanced tube-like structure formation on soft matrices, but did not significantly increase fibrogenesis on stiff matrices (Fig. 3M). Consistently, the expression of α-SMA on stiff matrices was not further elevated by PSPC1 overexpression (Fig. 3N and O). Notably, the mRNA levels of *Cdh5* and *Pdgfa* were significantly increased by PSPC1 overexpression with TGF-β1 treatment on soft matrices (Fig. 3P). These findings suggest that despite PSPC1 downregulation under soft matrix conditions, a minimal threshold of PSPC1 expression may still be critical for TGF-β1-induced tube-like structure formation and angiogenesis. On stiff matrices, however, PSPC1 expression may reach a threshold that is sufficient to interact with Smad2 and promote TGF-β1-induced fibrogenesis, so that further PSPC1 overexpression does not enhance this effect. In contrast, downregulation of *Neat1* did not affect TGF-β1-induced angiogenesis on soft matrices. Taken together, both *Neat1* and PSPC1 upregulated by TGF-β1 on stiff matrices promote MKPC transdifferentiation into myofibroblast, and the response of PSPC1 on soft matrices plays a role in TGF-β1-treated MKPC to undergo angiogenesis-related tubule formation.

### β1-integrin mechanotransduction regulates *Neat1* but not PSPC1 in response to matrix stiffness

**Fig. 4:**
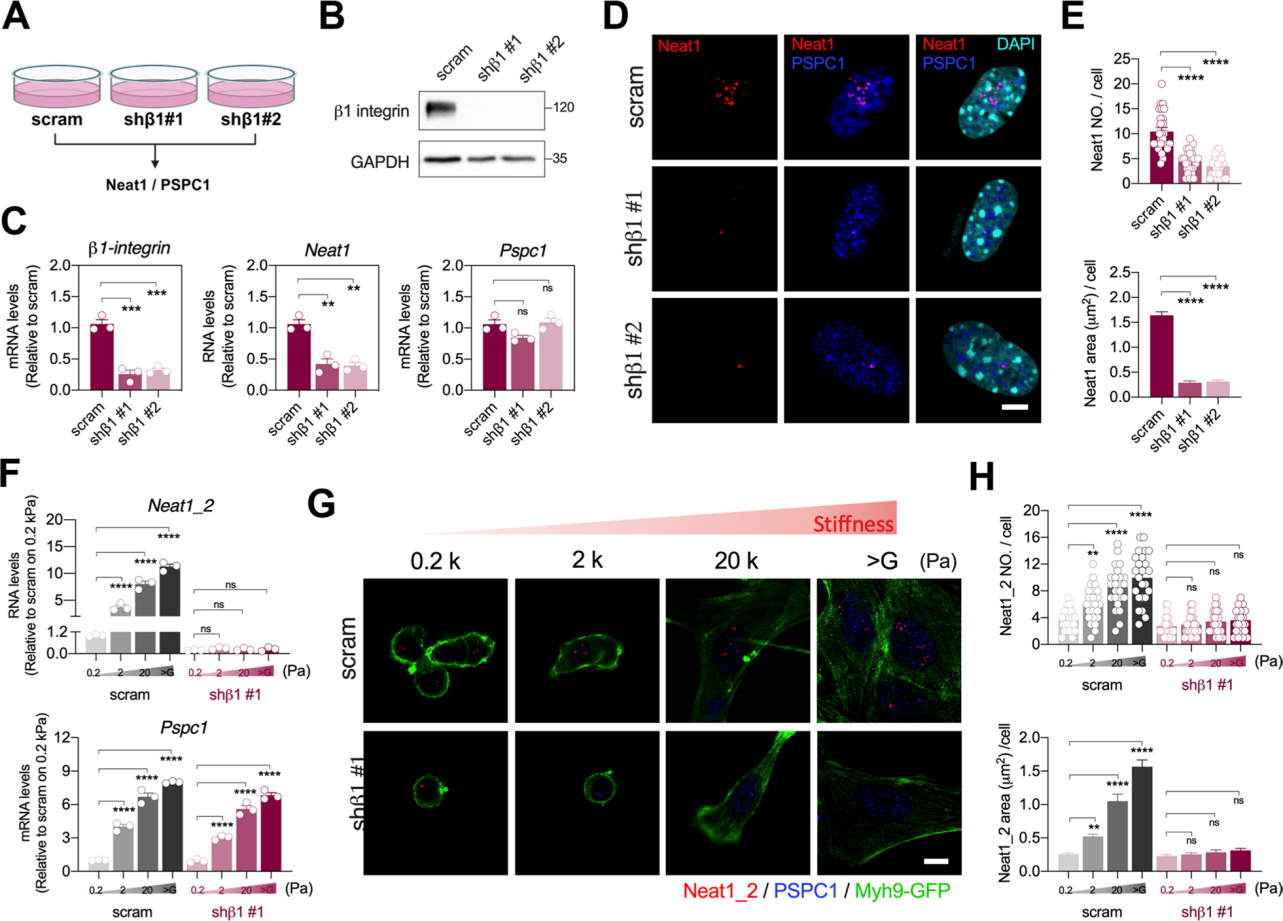
β1-integrin is required for *Neat1* upregulation and assembly on stiff matrices. **A** Diagram of the experimental design of MKPCs infected with shRNA of β1-intergrin (shβ1-intergrin stable clones #1 and #2) and analyzed for target gene expressions. **B** Representative Western blot images showing β1-integrin expression in MKPCs infected with scramble and shβ1-intergrin, using GAPDH as the internal control. **C** Gene expression of β1-integrin, *Neat1,* and *Pspc1* in MKPCs infected with shβ1-intergrin, analyzed by qRT-PCR. **D** Representative images of *in-situ* hybridization of *Neat1* (red) and immunofluorescence staining of PSPC1 (blue) in shβ1-intergrin-infected MKPCs on stiff matrix. Scale bar: 10 μm. **E** Quantification of the number and area of *Neat1* in shβ1-intergrin-infected MKPCs. **F** Gene expression of *Neat1_2*, and *Pspc1* in MKPCs infected with shβ1-intergrin, analyzed by qRT-PCR. **G** Representative images of *in-situ* hybridization of *Neat1* (red) and immunofluorescence staining of PSPC1 (blue) and Myh9-GFP (green) in shβ1-intergrin-infected MKPCs grown on polyacrylamide (PA) gels with stiffness levels of 0.2 kPa, 2 kPa, 20 kPa, and > G Pa. Scale bar: 20 μm. **H** Quantification of the number and area of *Neat1_2* per cell in MKPCs infected with shβ1-intergrin grown on matrices of various stiffness. At least 20 representative images were analyzed for each condition (**D**, **G**). Data are presented as individual points with mean ± S.E.M. from at least three independent experiments (**C**, **E**, **F**, **H**). Statistical analysis was performed using one-way ANOVA (C, E) and two-way ANOVA (F, H), with Bonferroni multiple comparison tests. *P < 0.05; **P < 0.01; ***P < 0.001; ****P < 0.0001

Environmental mechanical stimulation is sensed and transduced into the cell through focal adhesions, particularly via β1-integrin, a membrane receptor that connects the ECM to the intracellular focal adhesion complex [19, 20]. Mechanical signaling of collagen fibrils from β1-integrin activates focal adhesion kinase (FAK) and triggers various downstream mechanosensitive pathways, impacting cell behaviors such as proliferation, migration and invasion [21]. Notably, β1-integrin protein expression was stiffness-dependent, lower on soft matrices and higher on stiff matrices (Fig. 1F and G; Fig. S4A). Based on this, we hypothesized that β1-integrin is involved in regulating mechanosensitive paraspeckle components, thereby influencing the differentiation of MKPCs. To test this hypothesis, we knockdowned β1-integrin in MKPCs using shRNA to investigate whether β1-integrin plays a role in regulating matrix stiffness-modulated *Neat1* and PSPC1 expression (Fig. 4A). The knockdown efficiency of shβ1-integrin in MKPCs was validated at both protein and RNA levels (Fig. 4B and C). Interestingly, *Neat1* and *Neat1_2* levels were reduced in β1-integrin-knockdowned MKPCs, while the mRNA level of PSPC1 remained unchanged (Fig. 4C and Fig. S4B). Additionally, the number and size of *Neat1* and *Neat1_2* in the nucleus were diminished following β1-integrin knockdown on stiff matrices, revealing that β1-integrin-mediated transduction pathway was involved in regulation of *Neat1* levels but not PSPC1 (Fig. 4D and E; Fig. S4C and S4D).

To further validate whether β1-integrin regulates matrix stiffness-dependent *Neat1* and PSPC1 expression, β1-integrin-knockdowned MKPCs were cultured on monomeric Col-coated PA gels with varying stiffness. We found that knockdown of β1-integrin completely abolished *Neat1* expression, while having no effect on the *Pspc1* RNA level regardless of matrix rigidity (Fig. 4F). The number and size of *Neat1_2* on stiffer matrices (2 k, 20 k and > G Pa) were downregulated to a level similar to those on soft 0.2 k Pa gels (Fig. 4G and H). Blocking mechanotransduction on stiffer matrices using shβ1-integrin decreased *Neat1_2* assembly, resulting in a mechanical response comparable to that of soft matrices with weak mechanical simulation. These results showed that matrix stiffness-regulated *Neat1* expression, but not PSPC1 expression, is regulated through β1-integrin-dependent mechanotransduction.

### β1-integrin-mediated *Neat1* expression is dependent on YAP nuclear translocation

**Fig. 5:**
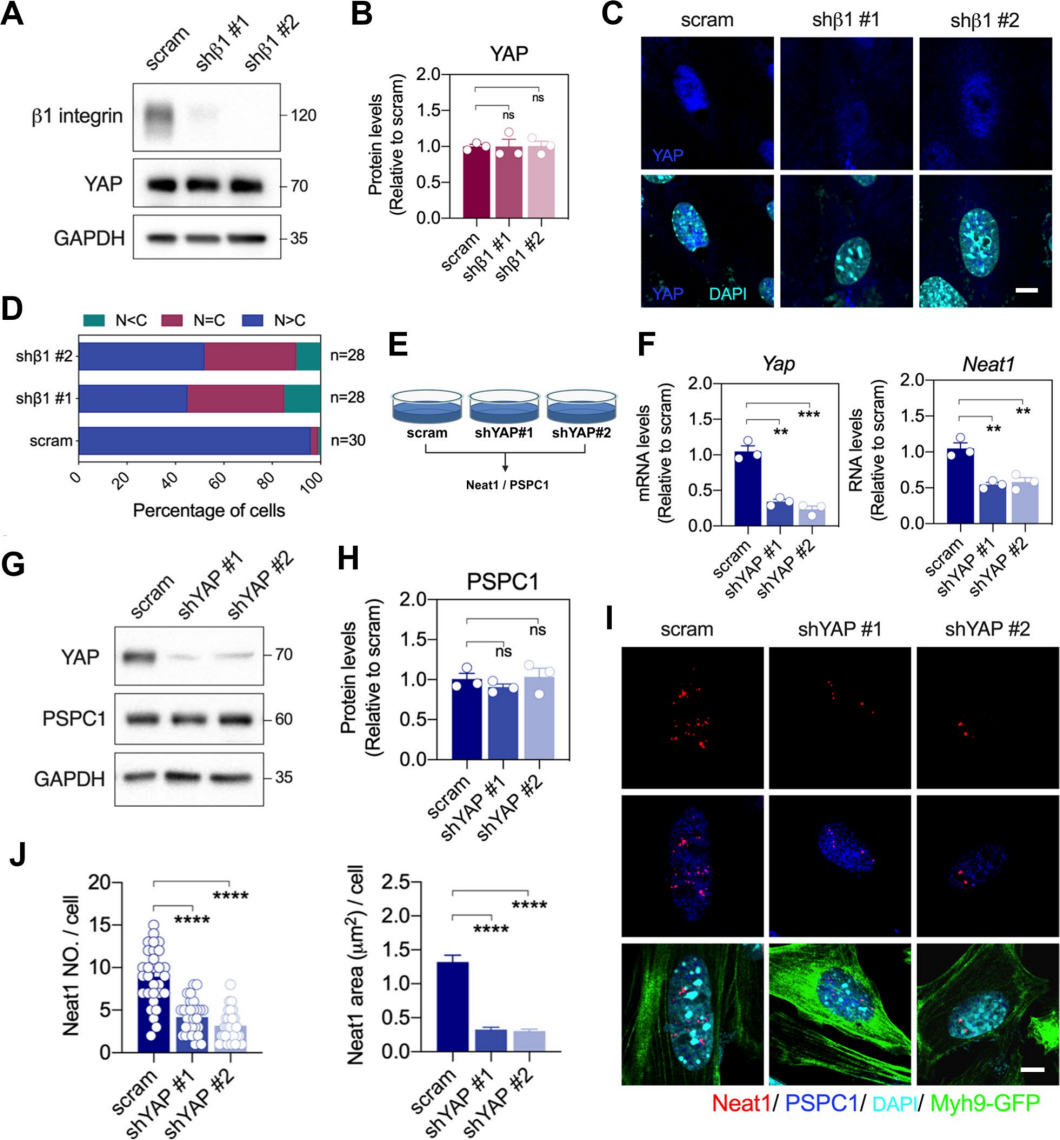
*Neat1* upregulation and assembly induced by stiff matrices were regulated by β1-integrin-dependent YAP nuclear localization. **A** Representative Western blot images of β1-integrin and YAP expressions in MKPCs infected with scramble and shβ1-intergrin, using GAPDH as the internal control. **B** Quantification of YAP expression in MKPCs infected with shβ1-intergrin. **C** Representative immunofluorescence images showing YAP (blue) and nuclei (cyan) on shβ1-intergrin-infected MKPCs on stiff matrix. Scale bar: 10 μm. **D** Percentage of the spatial distribution of YAP in the nuclear and cytosol in shβ1-intergrin-infected MKPCs. N: nuclear. C: cytosol. **E** Diagram of the experimental design of MKPCs treated with shYAP (stable clone #1 and #2) and analyzed for target gene expression. **F** Gene expression *Yap* and *Neat1* in MKPCs infected with shYAP, analyzed by qRT-PCR. **G** Representative Western blot images of YAP and PSPC1 in MKPCs infected with shYAP, using GAPDH as the internal control. **H** Quantification of PSPC1 protein expression in YAP-knockdowned MKPCs. **I** Representative images of *in-situ* hybridization of *Neat1* (red) and immunofluorescence staining of PSPC1 (blue) and Myh9-GFP (green) in shYAP-infected MKPCs on stiff matrix. **J** Quantification of the number and area of *Neat1* in shYAP-infected MKPCs. Data are presented as individual points with mean ± S.E.M. from at least three independent experiments (**B**, **F**, **G**, **J**). At least 20 representative images were analyzed for each condition (**C**, **I**). Statistical analysis was performed using one-way ANOVA (**B**, **F**, **H**, **J**) with Bonferroni multiple comparison tests. *P < 0.05; **P < 0.01; ***P < 0.001; ****P < 0.0001

One of the downstream mechanosensitive pathways of β1-integrin is the YAP/TAZ pathway, which relies on mechanical cues to promote YAP/TAZ nuclear translocation and facilitate its transcriptional function, regulating cellular behaviors such as proliferation and differentiation [22, 23]. As matrix stiffness increases, YAP nuclear translocation also increases [24–26]. To determine whether β1-integrin-regulated *Neat1* level was mediated by YAP, we examined YAP expression in β1-integrin-knockdowned MKPCs. Although knockdown of β1-integrin did not alter YAP protein expression on stiff matrices, a higher percentage of cells showed a low or equal N/C ratio of YAP compared to the control group, implying notably reduced YAP nuclear translocation (Fig. 5A‐D).

To examine whether YAP is involved in regulating *Neat1* expression, we depleted YAP in MKPCs using shRNA, and the reductions in YAP RNA and protein levels were confirmed (Fig. 5E‐G). We observed that knockdown of YAP led to downregulation of *Neat1*, but not PSPC1 (Fig. 5F‐H). The number and area of *Neat1* in the nucleus were significantly reduced by knockdown of YAP (Fig. 5I and J). Additionally, the downstream loci of YAP predicted by ChIP database included the *Neat1* gene loci (Fig. S6A). These findings support the notion that β1-integrin-mediated *Neat1* expression requires YAP nuclear localization for activation in response to matrix stiffness.

### Piezo1-mediated calcium influx is critical for the expression of *Neat1* and PSPC1

**Fig. 6:**
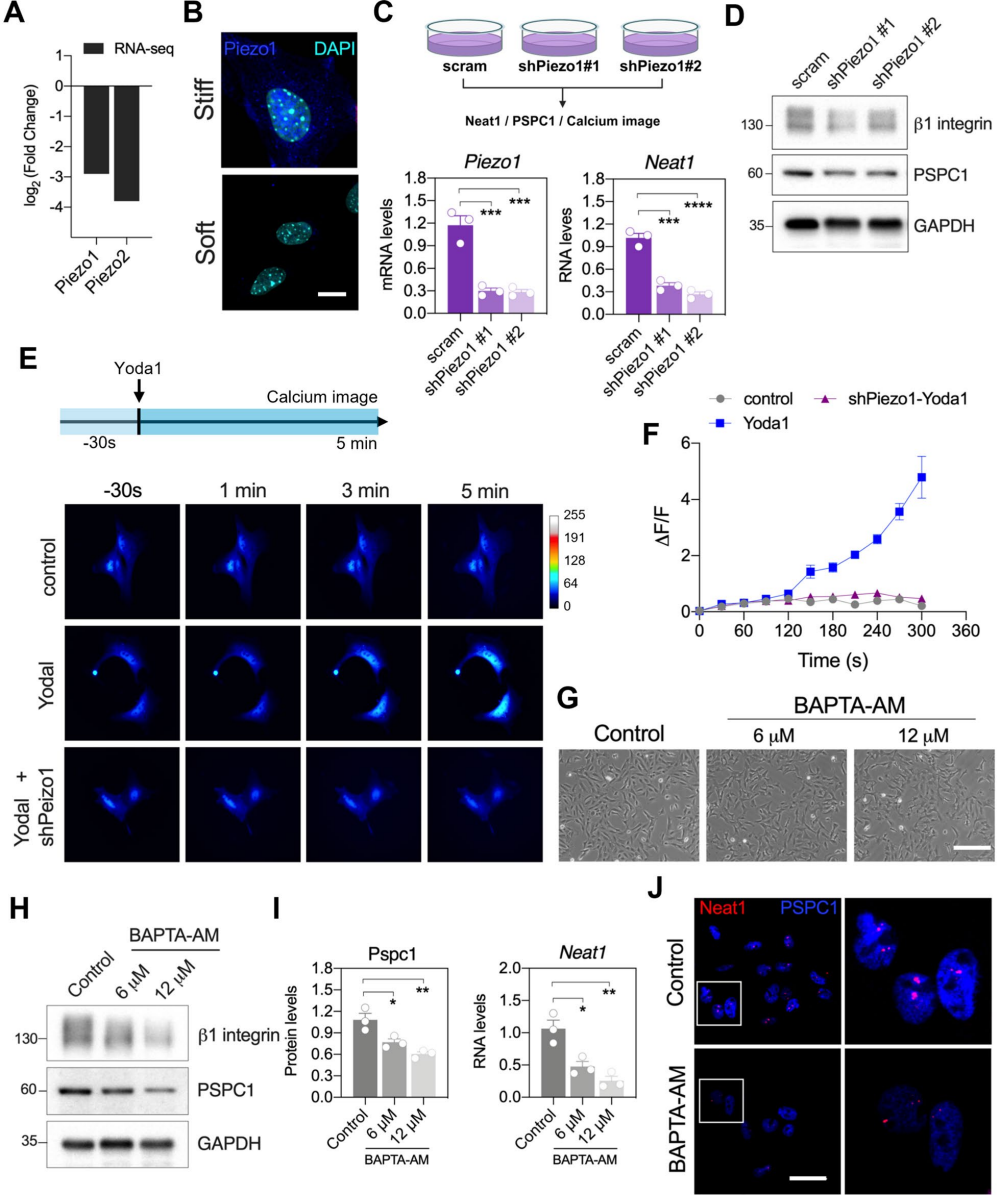
Piezo1-mediated intracellular calcium regulated *Neat1* and paraspeckle complexes. **A** RNA‑seq data showing the expression levels of *Piezo1* and *Piezo2* on a soft matrix versus a stiff matrix. **B** Representative images of Piezo1 (blue) and nuclei (cyan)in MKPCs on stiff and soft matrix. Scale bar: 10 μm. **C** The upper diagram showing the experimental design of MKPCs treated with shPiezo1 (stable clones #1 and #2). The lower panels showing the gene expressions of *Piezo1* and *Neat1* in MKPCs infected with shPiezo1, analyzed by qRT-PCR. **D** Representative Western blot images of β1-intergrin and PSPC1 in MKPCs infected with shPiezo1, using GAPDH as the internal control. **E** Time-lapse images of calcium influx measured by R-GECO1 in control and shPiezo1-infected MKPCs treated with Yoda1. **F** △F/F traces analysis showing the fluctuations of R-GECO1 fluorescence in control and shPiezo1-infected MKPCs treated with Yoda1. **G** Representative phase-contrast images of MKPCs treated with 6 and 12 µM BAPTA-AM. Scale bar: 200 μm. **H** Representative Western blot images showing β1-integrin and PSPC1 expressions in MKPCs treated with 6 and 12 µM BAPTA-AM, with GAPDH as the internal control. **I** Quantification of PSPC1 protein level and *Neat1* RNA level in MKPCs treated with BAPTA-AM. **J** Representative images of *in-situ* hybridization of *Neat1* (red) and immunofluorescence staining of PSPC1 (blue) in BAPTA-AM treated MKPCs. Scale bar: 30 μm. Data are presented as individual points with mean ± S.E.M. from at least three independent experiments (**C**, **F**, **I**). Statistical analysis was performed using one-way ANOVA (**C**, **I**) with Bonferroni multiple comparison tests. *P < 0.05; **P < 0.01; ***P < 0.001; ****P < 0.0001

Piezo1 and Piezo2 are mechanically activated calcium channels located on the cell membrane [27, 28]. The function of Piezo1 is associated with vascular structure and lymphatic dysplasia, while Piezo2 plays an important role in the neuronal transduction system [29–31]. During kidney fibrosis progression, Piezo1 plays a critical role in renal proximal tubular cells by activating the calcium/calpain2/β1-integrin pathway [32]. Thus, we wondered whether Piezo1 is involved in β1-integrin-YAP mediated *Neat1* or regulating PSPC1 expression. The RNA-seq data showed that the RNA levels of *Piezo1* and *Piezo2* were downregulated in MKPCs on soft matrices compared to stiff matrices (Fig. 6A). The RNA-seq results were further validated by immunofluorescence staining, which showed lower Piezo1 in MKPCs on soft matrices, demonstrating that Piezo1 expression was regulated by matrix stiffness (Fig. 6B).

To investigate whether Piezo1 regulates *Neat1* and PSPC1 in MKPCs, we knockdowned Piezo1 in MKPCs using shRNA and the knockdown efficacy was confirmed (Fig. 6C and Fig. S5A). Knockdown of Piezo1 led to a significant reduction in *Neat1* expression (Fig. 6C, *Neat1*). Moreover, the protein expressions of β1-integrin and PSPC1 were downregulated by shPiezo1 as well, suggesting that Piezo1 is not only required for β1-integrin-YAP-mediated-*Neat1* expression, but also for PSPC1 expression (Fig. 6C and D; Fig. S5B).

To explore whether knockdown or activation of Piezo1 modulates intracellular calcium concentration in MKPCs, we monitored the dynamic calcium influx in Piezo1-knockdowned MKPCs with selective Pizeo1 agonist Yoda1 using R-Geco1, an RFP-based calcium indicator (Fig. 6E) Administering Yoda1 to MKPCs significantly increased cytosolic calcium levels compared to the control vehicle (Fig. 6E and F). In contrast, Yoda1 failed to induce calcium influx in Piezo1-knockdown MKPCs, demonstrating that knockdown of Piezo1 in MKPCs effectively blocked the calcium influx and affected the mechanoresponses of *Neat1* and PSPC1 (Fig. 6E and F).

To investigate the influence of calcium signaling on *Neat1* and PSPC1 expressions, we validated the effective concentrations of the intracellular calcium chelator BAPTA-AM (Fig. 6G). BAPTA-AM decreased PSPC1 and β1-integrin expressions in a dose-dependent manner (Fig. 6H and I). Concurrently, the RNA level and immunofluorescence expression of *Neat1* were also reduced in MKPCs (Fig. 6I and J), indicating that calcium signaling is required for matrix stiffness-induced *Neat1* expression as well as PSPC1. These findings suggested that Piezo1-mediated intracellular calcium influx is the upstream of *Neat1* and paraspeckles in response to matrix stiffness.

## Discussion

Our research elucidates the mechanical mechanisms of the mechanical stimuli functions on determining TGF-β1-induced stem cell fates via mechanotransduction pathways, which was activated by cell membrane mechanoreceptors and transduced to nuclear mechanoresponders, *Neat1* and PSPC1. The responses of *Neat1* and PSPC1 to mechanical signals from outside of the cell were mediated through β1-integrin-YAP- and Piezo1-mediated pathways, changing their expression levels as reflected by the size and number of paraspeckle complexes (Fig. S6B). Our findings provide a new perspective on the mechanosensitive mechanisms involved in TGF-β1-induced SC specification in response to physical properties of the environment. In AKI, MSCs facilitate kidney regeneration in response to the soft matrix environment, whereas in CKD, MSCs promote fibrosis under TGF-β1 stimulation combined with elevated matrix stiffness. Our studies indicate that matrix stiffness plays a key role in determining the fate and function of MSCs during kidney injury.

Our study presents two key innovations: first, we demonstrated that matrix stiffness regulates the size and number of paraspeckle complex, whose contents play important roles in EMT via *Neat1* and PSPC1 respectively. Second, we observed that *Neat1* downregulation on stiff matrices with TGF-β1, consistent with release of PSPC1 from paraspeckles to exert its functional roles as transcriptional co-factors (Fig. 2C and Fig. S6C). Under stiff matrix stimulation, TGF-β1 induces Smad complex upregulation to interact with PSPC1 (Fig. S2), enhancing the expression of genes including EMT markers and myofibroblast activation [12]. This phenomenon was also observed in MKPC fate determination under TGF-β1 treatment on stiff matrices in our study (Fig. 1). In contrast, soft matrices reduced the expression levels of *Neat1* and PSPC1 (Fig. 2B and D). Although TGF-β1 reduced *Neat1* levels under soft matrices, low PSPC1 levels on soft matrices is insufficient for TGF-β1 to induce EMT changes. Instead, TGF-β1-induced pSmad signaling promotes MKPC differentiation into vascular endothelial cells on soft matrices. Our observations further reveal that the depletion of either PSPC1 or *Neat1* prevents TGF-β1-induced progenitor cell transdifferentiation into myofibroblasts and inhibits subsequent myofibroblast activation on stiff matrices. Notably, depletion of *Neat1* also alleviates TGF-β1-induced myofibroblast activation, though the underlying mechanisms remain to be investigated. Moreover, expression levels of *Neat1* and PSPC1 were markedly reduced in MKPCs cultured on soft collagen gels (Fig. 2B and D). Under such conditions, TGF-β1 treatment triggers gene expression of endothelial cells and angiogenesis, i.e. *Fgf2*, *Pdgfa*, *Vegfr2* and *Cd31* (Fig. 1K)*.* According to ChIP database analysis, pSmad3 binding sites can be frequently found in the sequences of these four genes (data not shown), providing a possible explanation for how soft matrix-induced downregulation of PSPC1 favors TGF-β1-induced angiogenesis in MKPC cells. However, further depletion of PSPC1 hindered TGF-β1-induced angiogenesis under soft conditions (Fig. 3K and L; Fig. S3C and S3D), suggesting that minimal levels of PSPC1 is required for endothelial cell fate determination.

In this study, we identified two distinct mechanosensory pathways that regulate paraspeckle complexes. The β1-integrin-YAP signaling activates *Neat1* expression, while the calcium signaling via the Piezo1 pathway mediates PSPC1 expression (Fig. S6B). Integrins function as mechanosensory receptors, converting mechanical signals into biochemical signals. They play a key role in cell adhesion to the ECM and regulate various cellular processes, including stem cell behaviors. Particularly, β1-integrin signaling in stem cells promotes self-renewal and maintenance of stemness by activating downstream signaling pathways such as FAK, Akt and YAP pathways [24, 33, 34]. YAP is a transcription factor that interacts with the transcriptional coactivator TAZ, which contains a PDZ-binding motif. YAP/TAZ shuttle dynamically between the nucleus and cytoplasm, modulating gene expression through binding with TEADs in the nucleus [35]. YAP/TAZ activity is regulated by extracellular ligands, such as growth factors, TGF-β1, hypoxic stress, and tissue stiffness [25, 36–41]. Recent studies highlight the crucial role of YAP/TAZ in various cellular processes, including cell survival, stem cell proliferation, cancer cell invasion, and organ fibrosis [23, 42–46]. Research further shows that a rigid matrix activates YAP activity, leading to its nuclear translocation and promoting the differentiation of MSCs into osteoblasts [25, 47]. Conversely, a soft matrix inhibits YAP/TAZ activity by restraining YAP/TAZ in the cytoplasm, thereby driving MSC differentiation into adipocytes [48, 49]. Here, we demonstrate for the first time that substrate stiffness triggers YAP nuclear import via a β1-integrin-dependent pathway in MKPCs, thereby activating *Neat1* transcription and promoting TGF-β1-induced transdifferentiation into myofibroblasts on a rigid matrix.

Piezo1 and Piezo2 ion channels have been implicated in mediating the effects of matrix stiffness on stem cell fate [50, 51]. Activation of Piezo1 and Piezo2 channels in response to mechanical cues triggers intracellular calcium signaling, which regulates downstream pathways associated with stem cell differentiation. Specifically, calcium influx through Piezo1 has been observed to stimulate osteogenic differentiation of MSCs on rigid substrates [52]. However, the precise mechanisms by which substrate stiffness regulate the Piezo family to mediate cellular functions remain unclear. Our research has identified that *Neat1* and PSPC1 play a critical role in TGF-β1-induced transdifferentiation of MKPCs into myofibroblasts on a rigid matrix (Fig. 3). This process occurs through a novel signaling pathway, where Piezo1, via calcium influx, modulates nuclear paraspeckle components (*Neat1* and PSPC1) in response to matrix rigidity (Fig. 6). Reduced levels of *Neat1* and PSPC1 prevent the differentiation of MKPCs into myofibroblasts under TGF-β1 stimulation on a soft matrix (Fig. 3 and Fig. S3). Therefore, dysregulation of these pathways may be linked to various disorders, including cancer, neurodegenerative diseases, and fibrosis conditions.

## Conclusions

Taken together, this study connects paraspeckle dynamics to extracellular mechanical cues in regulation of TGF-β1-driven MSC specification, emphasizing their involvement through β1-integrin-YAP and Piezo1 pathways. These findings offer valuable insights into the role of mechanotransduction in fibrosis development and may uncover novel therapeutic targets for its treatment.

## Supplementary Information

Supplementary file 1.

## Data Availability

The datasets used and/or analyzed during the current study are available from the corresponding author on reasonable request.

## Abbreviations

AKI: Acute kidney injury
CKD: Chronic kidney disease
SC: Stem cell
MSC: Mesenchymal stem cell
EMT: Epithelial mesenchymal transdifferentiation
PSPC1: Paraspeckle component 1
lncRNA: Long non-coding RNA
*Neat1*: Nuclear paraspeckle assembly transcript 1
MKPC: Mouse kidney progenitor cell
Col: Collagen
GPa: Gigapascal
PCA: Principle component analysis
ECM: Extracellular matrix
GO: Gene ontology
PA: Polyacrylamide
FAK: Focal adhesion kinase

## References

[1] Pavyde E, Usas A, Pockevicius A, Maciulaitis R. Muscle-derived stem/progenitor cells ameliorate acute kidney injury in rats through the anti-apoptotic pathway and demonstrate comparable effects to bone marrow mesenchymal stem cells. Medicina. 2023;60(1):63. 38256324 10.3390/medicina60010063 PMC10821316

[2] Gupta S, Verfaillie C, Chmielewski D, Kren S, Eidman K, Connaire J, et al. Isolation and characterization of kidney-derived stem cells. J Am Soc Nephrol. 2006;17(11):3028–40. 16988061 10.1681/ASN.2006030275

[3] Oh E, Wu H, Muto Y, Donnelly EL, Machado FG, Fan LX, et al. A conditionally immortalized Gli1-positive kidney mesenchymal cell line models myofibroblast transition. Am J Physiol Renal Physiol. 2019;316(1):63-F75. 10.1152/ajprenal.00460.2018 PMC6383201 30303712

[4] Wang YK, Wang YH, Wang CZ, Sung JM, Chiu WT, Lin SH, et al. Rigidity of collagen fibrils controls collagen gel-induced down-regulation of focal adhesion complex proteins mediated by alpha2beta1 integrin. J Biol Chem. 2003;278(24):21886–92. 12676963 10.1074/jbc.M300092200

[5] Wang YH, Chiu WT, Wang YK, Wu CC, Chen TL, Teng CF, et al. Deregulation of AP-1 proteins in collagen gel-induced epithelial cell apoptosis mediated by low substratum rigidity. J Biol Chem. 2007;282(1):752–63. 17085440 10.1074/jbc.M604801200

[6] Chen WC, Lin HH, Tang MJ. Matrix-stiffness-regulated inverse expression of Kruppel-Like factor 5 and Kruppel-Like factor 4 in the pathogenesis of renal fibrosis. Am J Pathol. 2015;185(9):2468–81. 26212907 10.1016/j.ajpath.2015.05.019

[7] Klein EA, Yin L, Kothapalli D, Castagnino P, Byfield FJ, Xu T, et al. Cell-cycle control by physiological matrix elasticity and in vivo tissue stiffening. Curr Biol. 2009;19(18):1511–8. 19765988 10.1016/j.cub.2009.07.069 PMC2755619

[8] Chen WC, Lin HH, Tang MJ. Regulation of proximal tubular cell differentiation and proliferation in primary culture by matrix stiffness and ECM components. Am J Physiol Renal Physiol. 2014;307(6):F695-707. 25056346 10.1152/ajprenal.00684.2013

[9] Sasaki YT, Ideue T, Sano M, Mituyama T, Hirose T. MENepsilon/beta noncoding RNAs are essential for structural integrity of nuclear paraspeckles. Proc Natl Acad Sci USA. 2009;106(8):2525–30. 19188602 10.1073/pnas.0807899106 PMC2650297

[10] Hirose T, Virnicchi G, Tanigawa A, Naganuma T, Li R, Kimura H, et al. NEAT1 long noncoding RNA regulates transcription via protein sequestration within subnuclear bodies. Mol Biol Cell. 2014;25(1):169–83. 24173718 10.1091/mbc.E13-09-0558 PMC3873887

[11] Fox AH, Nakagawa S, Hirose T, Bond CS. Paraspeckles: where long noncoding RNA meets phase separation. Trends Biochem Sci. 2018;43(2):124–35. 29289458 10.1016/j.tibs.2017.12.001

[12] Yeh HW, Hsu EC, Lee SS, Lang YD, Lin YC, Chang CY, et al. Pspc1 mediates TGF-beta1 autocrine signalling and Smad2/3 target switching to promote EMT, stemness and metastasis. Nat Cell Biol. 2018;20(4):479–91. 29593326 10.1038/s41556-018-0062-y

[13] Wang Y, Hu SB, Wang MR, Yao RW, Wu D, Yang L, et al. Genome-wide screening of NEAT1 regulators reveals cross-regulation between paraspeckles and mitochondria. Nat Cell Biol. 2018;20(10):1145–58. 30250064 10.1038/s41556-018-0204-2

[14] Liu C, Gao X, Li Y, Sun W, Xu Y, Tan Y, et al. The mechanosensitive lncRNA Neat1 promotes osteoblast function through paraspeckle-dependent Smurf1 mRNA retention. Bone Res. 2022;10(1):18. 35210394 10.1038/s41413-022-00191-3 PMC8873336

[15] Todorovski V, Fox AH, Choi YS. Matrix stiffness-sensitive long noncoding RNA NEAT1 seeded paraspeckles in cancer cells. Mol Biol Cell. 2020;31(16):1654–62. 32293985 10.1091/mbc.E20-02-0097 PMC7521846

[16] Lee PT, Lin HH, Jiang ST, Lu PJ, Chou KJ, Fang HC, et al. Mouse kidney progenitor cells accelerate renal regeneration and prolong survival after ischemic injury. Stem Cells. 2010;28(3):573–84. 20099318 10.1002/stem.310

[17] Chen CL, Chou KJ, Fang HC, Hsu CY, Huang WC, Huang CW, et al. Progenitor-like cells derived from mouse kidney protect against renal fibrosis in a remnant kidney model via decreased endothelial mesenchymal transition. Stem Cell Res Ther. 2015;6:239. 26631265 10.1186/s13287-015-0241-8 PMC4668678

[18] Kandow CE, Georges PC, Janmey PA, Beningo KA. Polyacrylamide hydrogels for cell mechanics: steps toward optimization and alternative uses. Methods Cell Biol. 2007;83:29–46. 17613303 10.1016/S0091-679X(07)83002-0

[19] Seetharaman S, Etienne-Manneville S. Integrin diversity brings specificity in mechanotransduction. Biol Cell. 2018;110(3):49–64. 29388220 10.1111/boc.201700060

[20] Chen W, Lou J, Evans EA, Zhu C. Observing force-regulated conformational changes and ligand dissociation from a single integrin on cells. J Cell Biol. 2012;199(3):497–512. 23109670 10.1083/jcb.201201091 PMC3483124

[21] Pang X, He X, Qiu Z, Zhang H, Xie R, Liu Z, et al. Targeting integrin pathways: mechanisms and advances in therapy. Signal Transduct Target Ther. 2023;8(1):1. 36588107 10.1038/s41392-022-01259-6 PMC9805914

[22] Elbediwy A, Vincent-Mistiaen ZI, Spencer-Dene B, Stone RK, Boeing S, Wculek SK, et al. Integrin signalling regulates YAP and TAZ to control skin homeostasis. Development. 2016;143(10):1674–87. 26989177 10.1242/dev.133728 PMC4874484

[23] Sabra H, Brunner M, Mandati V, Wehrle-Haller B, Lallemand D, Ribba AS, et al. β1 integrin–dependent Rac/group I PAK signaling mediates YAP activation of Yes-associated protein 1 (YAP1) via NF2/merlin. J Biol Chem. 2017;292(47):19179–97. 28972170 10.1074/jbc.M117.808063 PMC5702661

[24] Tan F, Huang Y, Pei Q, Liu H, Pei H, Zhu H. Matrix stiffness mediates stemness characteristics via activating the Yes-associated protein in colorectal cancer cells. J Cell Biochem. 2019;120(2):2213–25. 30218452 10.1002/jcb.27532

[25] Dupont S, Morsut L, Aragona M, Enzo E, Giulitti S, Cordenonsi M, et al. Role of YAP/TAZ in mechanotransduction. Nature. 2011;474(7350):179–83. 21654799 10.1038/nature10137

[26] Halder G, Dupont S, Piccolo S. Transduction of mechanical and cytoskeletal cues by YAP and TAZ. Nat Rev Mol Cell Biol. 2012;13(9):591–600. 22895435 10.1038/nrm3416

[27] Moroni M, Servin-Vences MR, Fleischer R, Sanchez-Carranza O, Lewin GR. Voltage gating of mechanosensitive PIEZO channels. Nat Commun. 2018;9(1):1096. 29545531 10.1038/s41467-018-03502-7 PMC5854696

[28] Coste B, Mathur J, Schmidt M, Earley TJ, Ranade S, Petrus MJ, et al. Piezo1 and Piezo2 are essential components of distinct mechanically activated cation channels. Science. 2010;330(6000):55–60. 20813920 10.1126/science.1193270 PMC3062430

[29] Li J, Hou B, Tumova S, Muraki K, Bruns A, Ludlow MJ, et al. Piezo1 integration of vascular architecture with physiological force. Nature. 2014;515(7526):279–82. 25119035 10.1038/nature13701 PMC4230887

[30] Lukacs V, Mathur J, Mao R, Bayrak-Toydemir P, Procter M, Cahalan SM, et al. Impaired PIEZO1 function in patients with a novel autosomal recessive congenital lymphatic dysplasia. Nat Commun. 2015;6:8329. 26387913 10.1038/ncomms9329 PMC4578306

[31] Ranade SS, Woo SH, Dubin AE, Moshourab RA, Wetzel C, Petrus M, et al. Piezo2 is the major transducer of mechanical forces for touch sensation in mice. Nature. 2014;516(7529):121–5. 25471886 10.1038/nature13980 PMC4380172

[32] Zhao X, Kong Y, Liang B, Xu J, Lin Y, Zhou N, et al. Mechanosensitive Piezo1 channels mediate renal fibrosis. JCI Insight. 2022. 10.1172/jci.insight.152330. 35230979 10.1172/jci.insight.152330 PMC9057604

[33] Vitillo L, Kimber SJ. Integrin and FAK regulation of human pluripotent stem cells. Curr Stem Cell Rep. 2017;3(4):358–65. 29177133 10.1007/s40778-017-0100-x PMC5683053

[34] Sheta M, Hassan G, Afify SM, Monzur S, Kumon K, Abu Quora HA, et al. Chronic exposure to FGF2 converts *i*PSCs into cancer stem cells with an enhanced integrin/focal adhesion/PI3K/AKT axis. Cancer Lett. 2021;521:142–54. 34455015 10.1016/j.canlet.2021.08.026

[35] Lin KC, Park HW, Guan KL. Regulation of the hippo pathway transcription factor TEAD. Trends Biochem Sci. 2017;42(11):862–72. 28964625 10.1016/j.tibs.2017.09.003 PMC5735856

[36] Chen J, Harris RC. Interaction of the EGF receptor and the hippo pathway in the diabetic kidney. J Am Soc Nephrol. 2016;27(6):1689–700. 26453611 10.1681/ASN.2015040415 PMC4884112

[37] Yu FX, Zhao B, Panupinthu N, Jewell JL, Lian I, Wang LH, et al. Regulation of the Hippo-YAP pathway by G-protein-coupled receptor signaling. Cell. 2012;150(4):780–91. 22863277 10.1016/j.cell.2012.06.037 PMC3433174

[38] Miranda MZ, Bialik JF, Speight P, Dan Q, Yeung T, Szaszi K, et al. TGF-beta1 regulates the expression and transcriptional activity of TAZ protein via a Smad3-independent, myocardin-related transcription factor-mediated mechanism. J Biol Chem. 2017;292(36):14902–20. 28739802 10.1074/jbc.M117.780502 PMC5592669

[39] Ma B, Chen Y, Chen L, Cheng H, Mu C, Li J, et al. Hypoxia regulates Hippo signalling through the SIAH2 ubiquitin E3 ligase. Nat Cell Biol. 2015;17(1):95–103. 25438054 10.1038/ncb3073

[40] Moon S, Kim W, Kim S, Kim Y, Song Y, Bilousov O, et al. Phosphorylation by NLK inhibits YAP-14-3-3-interactions and induces its nuclear localization. EMBO Rep. 2017;18(1):61–71. 27979972 10.15252/embr.201642683 PMC5210122

[41] Aragona M, Panciera T, Manfrin A, Giulitti S, Michielin F, Elvassore N, et al. A mechanical checkpoint controls multicellular growth through YAP/TAZ regulation by actin-processing factors. Cell. 2013;154(5):1047–59. 23954413 10.1016/j.cell.2013.07.042

[42] Chang HA, Ou Yang RZ, Su JM, Nguyen TMH, Sung JM, Tang MJ, et al. YAP nuclear translocation induced by HIF-1alpha prevents DNA damage under hypoxic conditions. Cell Death Discov. 2023;9(1):385. 37863897 10.1038/s41420-023-01687-5 PMC10589224

[43] Panciera T, Azzolin L, Fujimura A, Di Biagio D, Frasson C, Bresolin S, et al. Induction of expandable tissue-specific stem/progenitor cells through transient expression of YAP/TAZ. Cell Stem Cell. 2016;19(6):725–37. 27641305 10.1016/j.stem.2016.08.009 PMC5145813

[44] Illes B, Fuchs A, Gegenfurtner F, Ploetz E, Zahler S, Vollmar AM, et al. Spatio-selective activation of nuclear translocation of YAP with light directs invasion of cancer cell spheroids. iScience. 2021;24(3):102185. 33718837 10.1016/j.isci.2021.102185 PMC7921841

[45] He X, Tolosa MF, Zhang T, Goru SK, Ulloa Severino L, Misra PS, et al. Myofibroblast YAP/TAZ activation is a key step in organ fibrogenesis. JCI Insight. 2022. 10.1172/jci.insight.146243. 35191398 10.1172/jci.insight.146243 PMC8876427

[46] Garoffolo G, Casaburo M, Amadeo F, Salvi M, Bernava G, Piacentini L, et al. Reduction of cardiac fibrosis by interference with YAP-dependent transactivation. Circ Res. 2022;131(3):239–57. 35770662 10.1161/CIRCRESAHA.121.319373

[47] Hsiao C, Lampe M, Nillasithanukroh S, Han W, Lian X, Palecek SP. Human pluripotent stem cell culture density modulates YAP signaling. Biotechnol J. 2016;11(5):662–75. 26766309 10.1002/biot.201500374 PMC4850094

[48] Olivares-Navarrete R, Lee EM, Smith K, Hyzy SL, Doroudi M, Williams JK, et al. Substrate stiffness controls osteoblastic and chondrocytic differentiation of mesenchymal stem cells without exogenous stimuli. PLoS ONE. 2017;12(1):e0170312. 28095466 10.1371/journal.pone.0170312 PMC5240960

[49] Karystinou A, Roelofs AJ, Neve A, Cantatore FP, Wackerhage H, De Bari C. Yes-associated protein (YAP) is a negative regulator of chondrogenesis in mesenchymal stem cells. Arthritis Res Ther. 2015;17(1):147. 26025096 10.1186/s13075-015-0639-9 PMC4449558

[50] Sugimoto A, Miyazaki A, Kawarabayashi K, Shono M, Akazawa Y, Hasegawa T, et al. Piezo type mechanosensitive ion channel component 1 functions as a regulator of the cell fate determination of mesenchymal stem cells. Sci Rep. 2017;7(1):17696. 29255201 10.1038/s41598-017-18089-0 PMC5735093

[51] Qiu X, Deng Z, Wang M, Feng Y, Bi L, Li L. Piezo protein determines stem cell fate by transmitting mechanical signals. Hum Cell. 2023;36(2):540–53. 36580272 10.1007/s13577-022-00853-8

[52] Xing Y, Yang B, He Y, Xie B, Zhao T, Chen J. Effects of mechanosensitive ion channel Piezo1 on proliferation and osteogenic differentiation of human dental follicle cells. Ann Anat. 2022;239:151847. 34687906 10.1016/j.aanat.2021.151847

