# Supplementary material for: Regulation of the mechanoresponsive Neat1 and PSPC1 by substrate stiffness in TGF-β1-induced renal progenitor cell fate
Source: J Biomed Sci. 2025 Nov 17;32:99. doi: 10.1186/s12929-025-01196-w (PMC12621399; doi:10.1186/s12929-025-01196-w)
Supplement: Supplementary file 1 [file 12929_2025_1196_MOESM1_ESM.docx]

**Supplementary Figures**


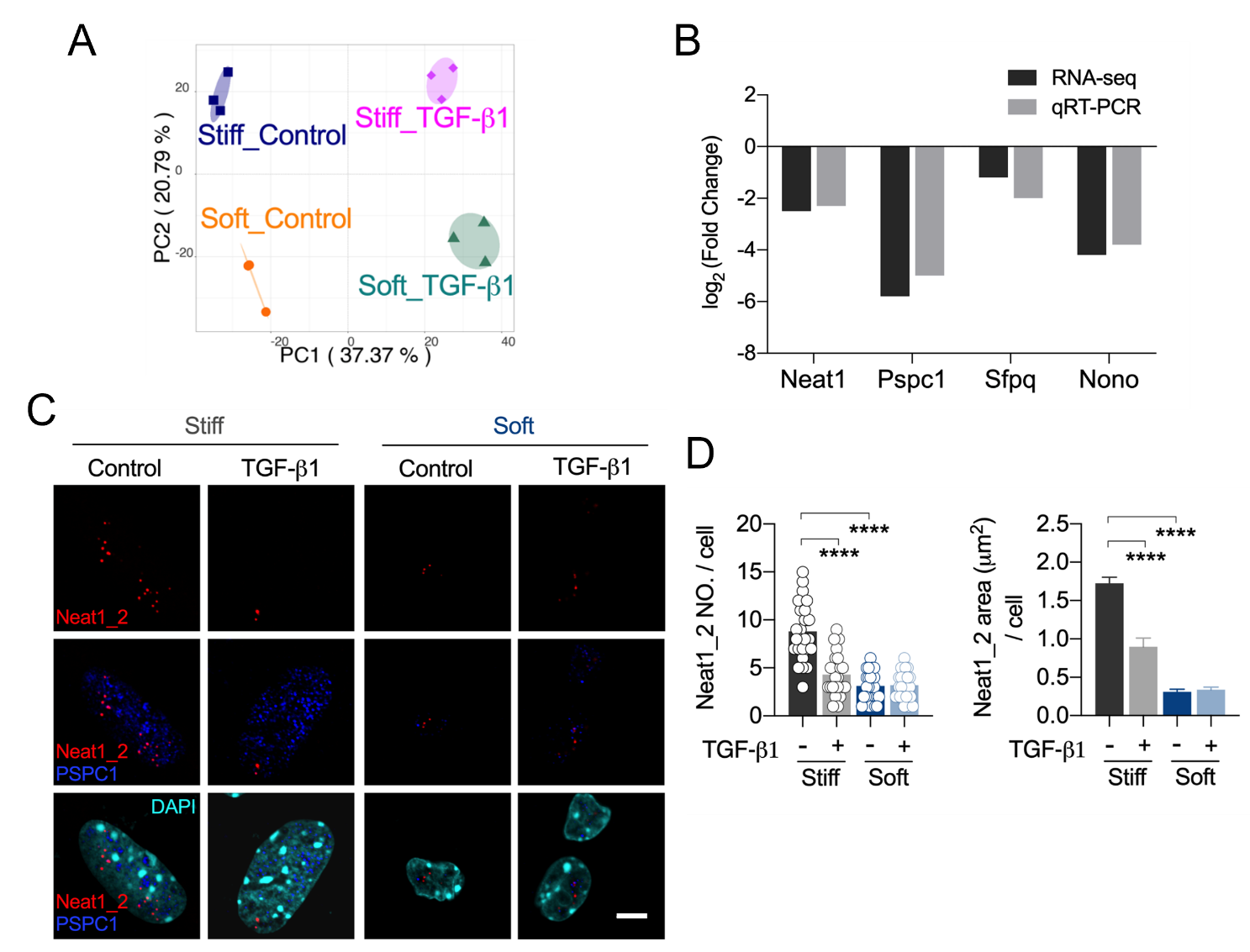


**Figure S1. Low stiffness of collagen gel decreased the expression levels of *Neat1_2*.**

**(A)** Principal component analysis (PCA) of gene expressions dataset from RNA-seq in control and TGF-β1-treated MKPCs on stiff or soft matrices. **(B)** A comparison between RNA‑seq data and RT‑qPCR results for lncRNAs and mRNAs on a soft matrix versus a stiff matrix. The validation results confirmed that the RNA‑seq data aligned well with the RT‑qPCR results. *GAPDH* gene was used as the internal reference control gene. **(C)** Representative images of in situ hybridization of *Neat1_2* (red) and immunofluorescence staining of PSPC1 (blue) and nuclei (cyan) on stiff and soft matrices with TGF-β1 treatment . Scale bar: 5 μm. **(D)** Quantification results showed the number and area of *Neat1_2* in cells grown on stiff and soft matrices with TGF-β1 treatment. Data are presented as individual points with mean ± S.E.M. from at least three independent experiments. Statistical analysis was performed using two-way ANOVA with Bonferroni multiple comparison tests. **P* < 0.05; ***P* < 0.01; ****P* < 0.001; *****P* < 0.0001.


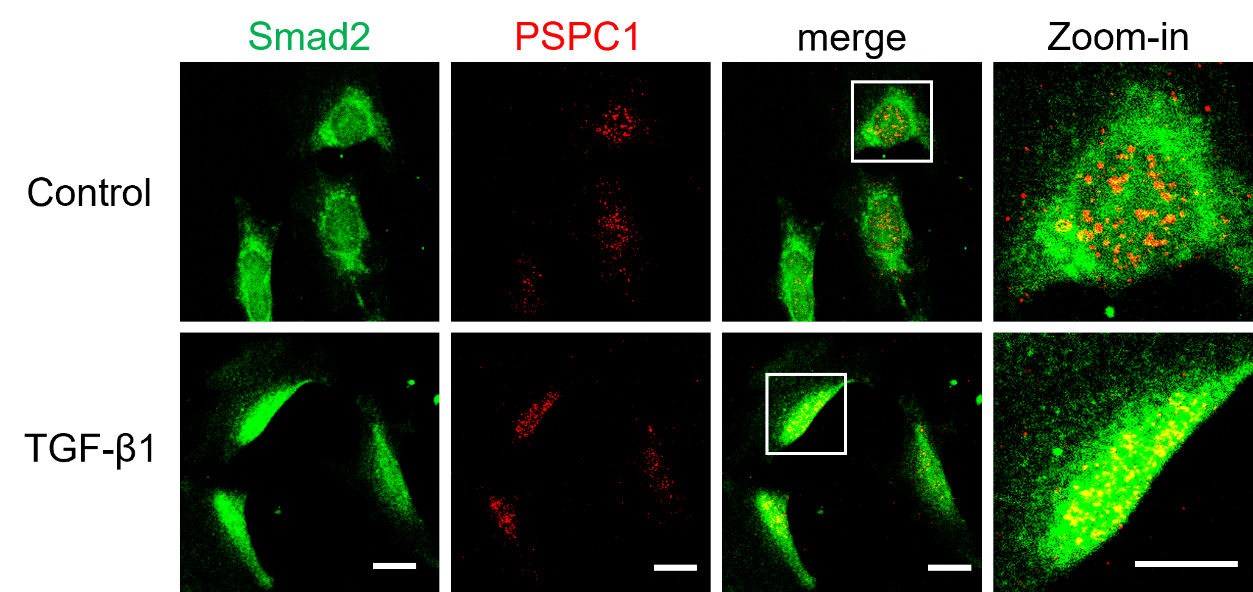


**Figure S2. PSPC1 colocalized with TGF-β1-induced nuclear Smad2 under stiff matrix condition.**

Representative immunofluorescence images of Smad2 (green) and PSPC1 (red) localization in cells cultured on stiff matrices upon TGF-β1 treatment. Scale bars: 15 μm (main images) and 10 μm (zoom-in).

**Figure S3. Knockdown of *Neat1*did not affect, but knockdown of PSPC1 reduced TGF-β1 induced expression of angiogenesis markers on soft matrices.**

**(A)** Gene expression analysis of endothelial markers *Cdh5* and *Vegfr2* and angiogenesis-related marer *Pdgfa* in siControl- and si*Neat1*-treated MKPCs on a soft matrix with TGF-β1 treatment. **(B)** Gene expression of the angiogenesis marker *Pdgfa* in siControl- and si*Neat1*-treated MKPCs on a soft matrix with TGF-β1 treatment. **(C-D)** Gene expression analysis of endothelial markers (*Cdh5* and *Vegfr2*) and the angiogenesis marker (*Pdgfa*). Data are presented as individual points with mean ± S.E.M. from at least three independent experiments. Statistical analysis was performed using two-way ANOVA with Bonferroni multiple comparison tests. ***P* < 0.01; ****P* < 0.001; *****P* < 0.0001.


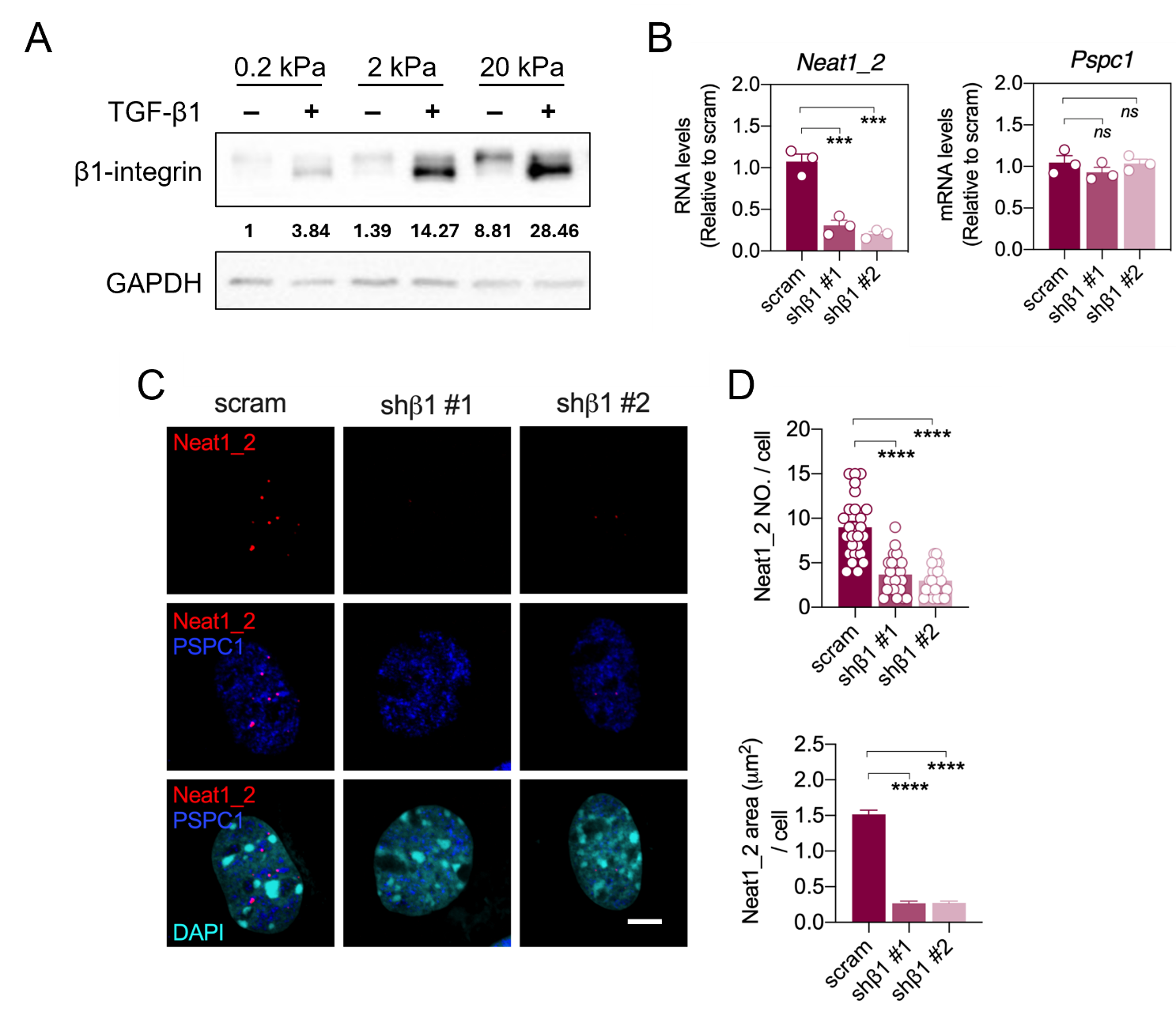


**Figure S4. β1-integrin was mechanosensitive and *Neat1_2* was regulated by β1-integrin-dependent pathway**

**(A)** Representative Western blot images showing β1-integrin protein levels in MKPCs cultured on matrices with varying stiffness: 0.2 kPa, 2 kPa and 20 kPa with TGF-β1 treatment. GAPDH is used as internal control. **(B)** Gene expression analysis of *Neat1_2* and *Pspc1* in MKPCs infected with shβ1-intergrin, analyzed by qRT-PCR. Gene expressions were normalized to *Gapdh*. **(C)** Representative images of *in-situ* hybridization showing *Neat1_2* (red) and immunofluorescence staining of PSPC1 (blue), and nuclei (cyan) on stiff matrices. Scale bar: 5 μm. **(D)** Quantification results showed the number and area of *Neat1_2* in cells grown on stiff matrices. Statistical analysis was performed using one-way ANOVA with Bonferroni multiple comparison tests. **P* < 0.05; ***P* < 0.01; ****P* < 0.001; *****P* < 0.0001.


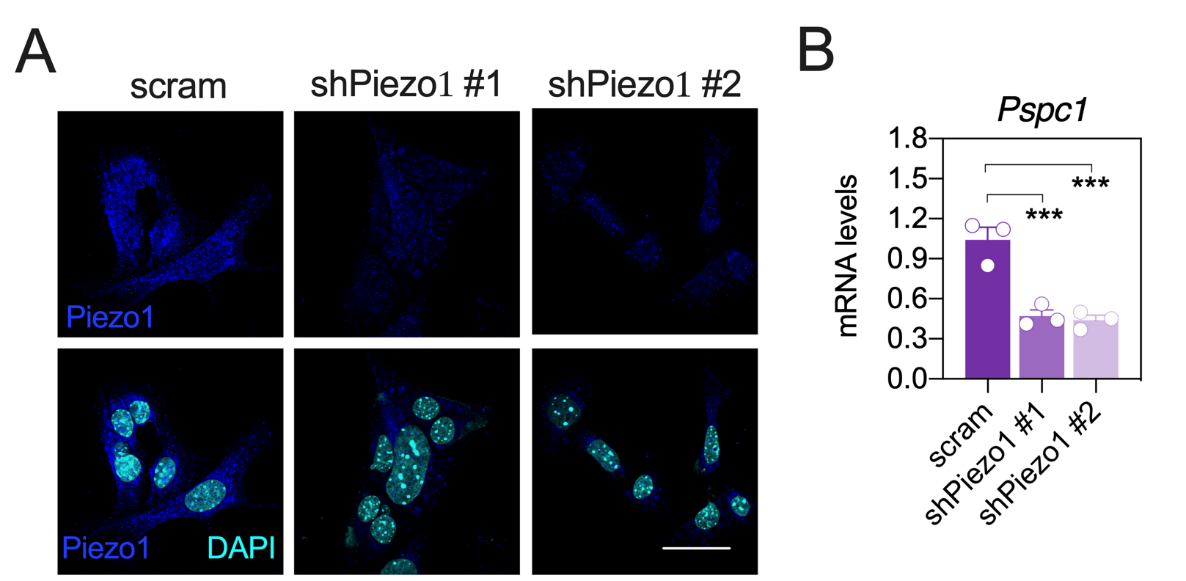


**Figure S5. Piezo1 is required for PSPC1 expression.**

**(A)** Representative immunofluorescence images of Piezo1 (blue) and nuclei (cyan) in MKPCs infected with shPiezo1. Scale bar: 30 μm. **(B)** Gene expression analysis of *Pspc1* RNA in MKPCs infected with shPiezo1, analyzed by qRT-PCR. Gene expressions were normalized to *Gapdh*. Data are presented as individual points with mean ± S.E.M. from at least three independent experiments. Statistical analysis was performed using one-way ANOVA with Bonferroni multiple comparison tests. **P* < 0.05; ***P* < 0.01; ****P* < 0.001; *****P* < 0.0001.


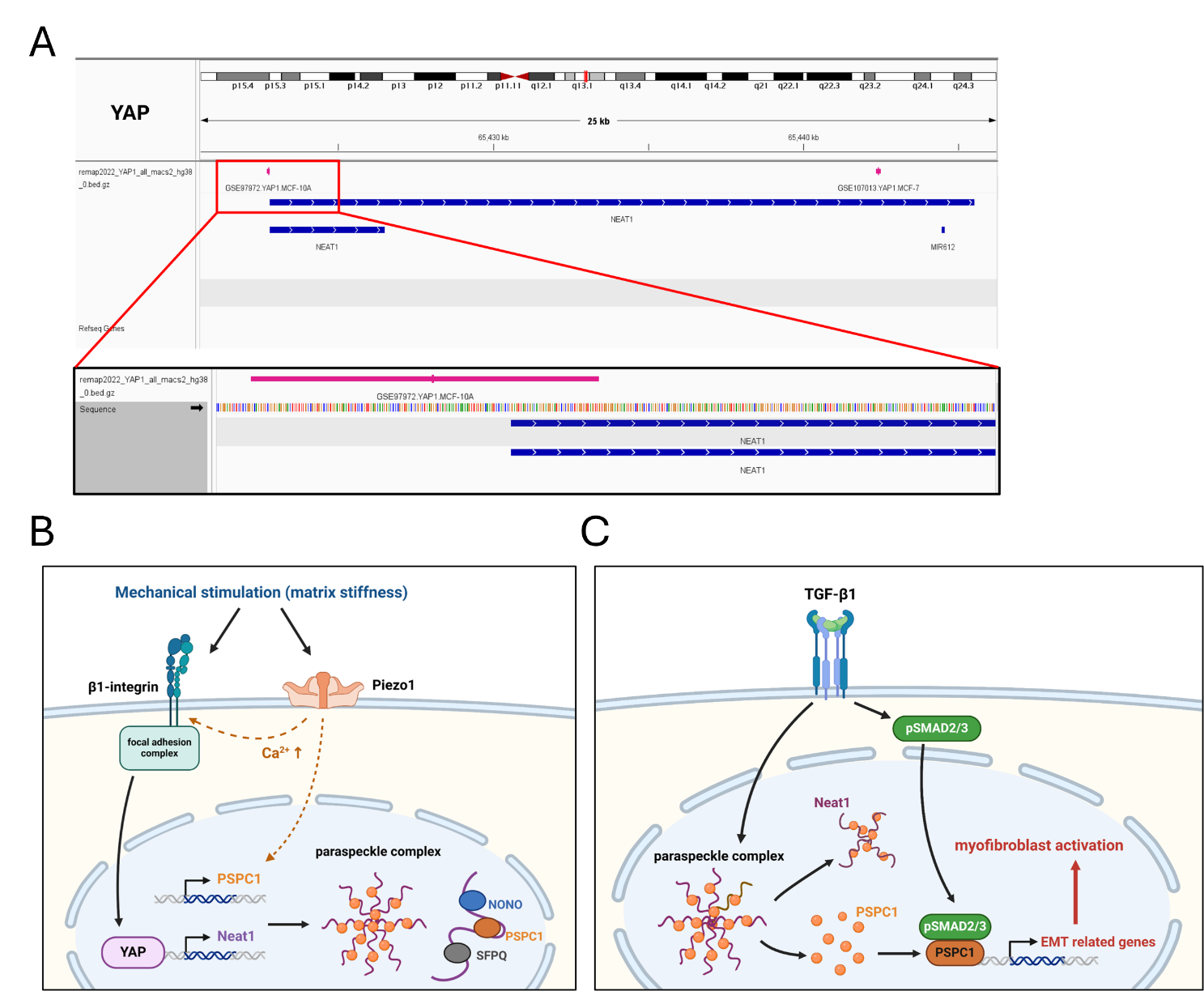


**Figure S6. Schematic illustrations of β1-integrin/Piezo1-paraspeckle and TGF-β1-paraspeckle signaling pathways in myofibroblast activation.**

**(A)** The genomic binding positions of YAP for the lncRNA *Neat1* gene loci were predicted using the ReMap database ([https://remap.univ-amu.fr](https://remap.univ-amu.fr/)) and visualized with IGV (Integrative Genomics Viewer). Pink regions indicate the YAP binding sites, and the blue regions represent the gene locus of *Neat1*. The shorter isoform (3756 bp) refers to *Neat1*, and the longer isoform (22743 bp) refers to *Neat1_2*. **(B)** Schematic illustration of the mechanical stimulation (stiff matrix)-β1-integrin/Piezo1-YAP-paraspeckle complex signaling pathway. **(C)** The signaling transduction pathways of TGF-β1-induced myofibroblast activation under stiff conditions. TGF-β1 decreases *Neat1* levels, which results in the release of PSPC1 from paraspeckle complex to facilitate the interactions with pSMAD2/3. Such interactions lead to the upregulation of gene expression of EMT and ultimately promote myofibroblast activation. All schematic illustrations were generated using Biorender.com.

**Table S1. The sequences of short hairpin RNA (shRNA) used in this article.**

| shRNA name | Clone ID | Target sequence (5’-3’) |
| --- | --- | --- |
| shPSPC1#1 | TRCN0000102472 | GCTAGACATGAACACCAGTTA |
| shPSPC1#2 | TRCN0000102473 | CTAGACATGAACACCAGTTAA |
| shβ1-intergrin#1 | TRCN0000066645 | GCCATTACTATGATTATCCTT |
| shβ1-intergrin#2 | TRCN0000066647 | GCACGATGTGATGATTTAGAA |
| shYAP#1 | TRCN0000238432 | GAAGCGCTGAGTTCCGAAATC |
| shYAP#2 | TRCN0000238436 | TGAGAACAATGACAACCAATA |
| shPiezo1#1 | TRCN0000141714 | GCTGCTCTGCTACTTCATCAT |
| shPiezo1#2 | TRCN0000142281 | GCACTCCATTATGTTCGAGGA |
